# New perspectives, additions, and amendments to plant endemism in a North African flora

**DOI:** 10.1186/s40529-024-00428-w

**Published:** 2024-07-16

**Authors:** Monier Abd El-Ghani, Hasnaa Hosni, Eman Shamso, Faten Ellmouni

**Affiliations:** 1 Botany and Microbiology Department, Faculty of Science, Cairo University, Giza, 12613 Egypt; 2 Botany Department, Faculty of Science, Fayoum University, Fayoum, 63514 Egypt

**Keywords:** Arid environment, Distribution patterns, Habitat diversity, Quantitative phytogeography, Egypt, Conservation, Rare taxa

## Abstract

**Background:**

Endemism is essential in biodiversity, biogeography, and conservation tasks. Based on herbarium specimens kept in some local herbaria, many published literature, and available information, we compiled a comprehensive list and an updated assessment of the Egyptian endemic and near-endemic taxa. The application of quantitative approaches to the distribution patterns, conservation status, and habitat preference of endemic taxa in Egypt was provided. Comparisons of the near-endemic taxa with other neighbouring flora were explained. For each taxon, the distribution patterns, most preferable habitat, biological spectrum, and taxa among 14 phytogeographical regions (Operational Geographical Units; OGUs) of Egypt were determined.

**Results:**

In this study, 19 endemics (out of 70) and 76 near-endemics (out of 181) are newly added taxa. Differentiation indices represented the taxonomic degrees of differentiation among endemic taxa. Two different indices were used to assess endemism: single-region endemic taxa (SRET) and multiple-region endemic taxa (MRET). Most endemic and near-endemic taxa were recorded from the mountainous Sinai (S) and the Mareotis sector of the Mediterranean coastal land (Mm). Generally, the most represented families in endemic and near-endemic areas were Asteraceae, Caryophyllaceae, Lamiaceae, and Fabaceae. More than 60% of the endemic taxa occurred in the sandy plains, wadis (desert valleys), and rocky plains and mountains. Applying hierarchical cluster analysis to the occurrences of 70 endemic taxa in the 14 studied OGUs revealed five main floristic groups (I–V), each characterized by certain OGUs. We provided eight groups of near-endemic taxa that represented their extension in neighbouring countries.

**Conclusions:**

The presented data will help to fill the gap in our knowledge of endemism, provide baseline information to understand biogeographical processes and facilitate further cooperation toward conservation purposes.

**Supplementary Information:**

The online version contains supplementary material available at 10.1186/s40529-024-00428-w.

## Introduction

The main floristic features in most countries are determined by specific components of endemic taxa, which play an essential role in biodiversity (Estill and Cruzan 2001) and conservation systems (Fois et al. 2017). The focus of biogeographic research is endemism (Crisp et al. 2001; Huang et al. 2011). Assuming that all individuals of a taxon are restricted to a given area or habitat, pure endemic taxa can be classified based on evolutionary history (neo- and paleo-endemics) or habitat specificity (Kruckeberg and Rabinowitz 1985; Ferreira and Boldrini 2011). Considering the size and limits of that area (Ladle and Whittaker 2011), endemic taxa may be local (limited to a small area), provincial (restricted to the confines of a province), national (limited to a country’s borders), regional (restricted to a geographical region), and continental (confined to a single continent). Endemism, which performs biogeographical characteristics of endemic taxa, refers to taxa limited to a specific geographic region or kind of environment or have short distribution ranges (Anderson 1994). Because endemic taxa are unevenly distributed worldwide, some areas could be better in such taxa, whereas others include high numbers. The loss of natural habitats, high sensitivity to human disturbance, and environmental changes may be attributed to the extinction of endemic plants (Thomas et al. 2004; Borokini 2014; Abdelaal et al. 2018). Although most of these taxa are believed to be a severe threat to biodiversity, they are listed on the Red Data List. A taxon with few records outside its target location is considered to have near-endemism (Noroozi et al. 2019). Certain dispersal events and temporary establishment in different habitats have resulted in near-endemic species (Matthews et al. 1993). Studying the distribution patterns of endemic taxa is a core issue in priorities for biodiversity conservation, such as important plant areas (IPA; Darbyshire et al. 2017), key biodiversity areas (KBA; IUCN 2012), and the criteria for zero-extinction sites (ZES; Ricketts et al. 2005; http://zeroextinction.org/the-alliance/about-the-alliance/).

Worldwide, the significant role of endemic plants in biodiversity has been addressed by various studies to fully understand and assess (qualitatively and/or quantitatively) this relationship. Crisp et al. (2001) focused on the character of environmental factors in the distribution patterns of endemism in Australian flora, Van Der Werff and Consiglio (2004) provided insights into the role of elevational gradients and biological spectrum for the distribution patterns of endemics in Peru, Figueiredo et al. (2009) presented the first account on the diversity and endemism in the Flora of Angola, Kallimanis et al. (2010) indicated the strong correlation between endemic taxa richness and biogeographical variables on islands of the Aegean archipelago, Huang et al. (2011) supplied the most thorough list of endemic Chinese seed plants together with an overview of their fundamental components and characteristics of distribution, Darbyshire et al. (2019) reviewed the diversity of endemic and near-endemic taxa in Mozambique that helped in identifying the Important Plant Areas (IPA), Demissew et al. (2021) discussed the future perspectives of local diversity and plant endemism in Ethiopia and Eritrea, Mehrabian et al. (2021) determined the Areas of Endemism (AOE) based on the distribution patterns and precedence for conservation of (endemic Iranian Monocots).

Applying a biogeographical approach to endemism proved helpful in determining the distribution patterns and diversity of endemic taxa. Rosser and Eggleton (2012) indicated that higher-rank taxa (e.g., genera and families) could be used successfully as alternatives to species richness, and almost all reports demonstrated that genus richness, as opposed to family or order richness, is a better substitute for species richness. Clustering techniques have been widely employed in biogeography and for determining endemic-rich regions where conservation activities should be implemented. These techniques are based on environmental variables and the distribution of endemic taxa. Médail et al. (1998) and Verlaque et al. (2001) reported the significant role of plant life forms in conservation and the impacts of global climate change.

Located in the north-eastern corner of the African Sahara, Egypt (between latitudes 22° and 32° N and longitudes 25° and 37° E) is the crossroads between Africa and Asia and adjoins the Mediterranean basin. The River Nile and the Red Sea are the two main corridors that link Egypt with tropical and equatorial Africa and the tropical Indian Ocean. Therefore, its flora of about 2145 species and 220 infraspecific taxa (Boulos 2009), is unique and comprises taxa of the Saharo-Arabian, Sudano-Zambezia, Irano-Turanian, and Mediterranean phytogeographical regions. Egypt has a hyper-arid environment (Abd El-Ghani et al. 2017a), which is reflected by harsh environmental conditions (Hegazy and Lovett-Doust 2016), with scanty rainfall (50–100 mm year^−1^) and high temperatures (Zahran and Willis 2009). Desert (Eastern, Western and Sinai) is the prevailing landscape comprising more than 90% of the total area (1 million km^2^). Consequently, desert vegetation, mainly composed of xerophytic shrubs and subshrubs, is the dominant natural plant life (Salama et al. 2016).

Over the past decades, several approaches have been applied to study the endemic and near-endemic taxa in the flora of Egypt. Täckholm (1974) and Boulos (1995, 2009) enumerated these taxa and confirmed their locations. The scattered published works between 2013 and 2021 (Hosni et al. 2013; Shaltout et al. 2018; Abdelaal et al. 2018, 2020; El-Khalafy et al. 2021) contributed significantly to the increase and improvement of our information on this group of species. The number of endemic taxa in Egyptian flora was controversial for a long time. Sixty-nine taxa were enumerated by Täckholm (1974), 60 by Boulos (2009), 76 by Hosni et al. (2013), 48 by Abdelaal et al. (2018), 140 by Abedelaal et al. (2020) and 41 by El-Khalafy et al. (2021). This number incongruence can be attributed to updated taxonomy and updated distribution information. Therefore, a final checklist of endemic and near-endemic taxa in Egypt is urgently recommended.

This study thoroughly updates other surveys conducted during the last decades. The application of quantitative approaches to the distribution patterns, conservation status, and habitat preference of endemic taxa in Egypt is assessed. Comparisons of the near-endemic taxa with other neighbouring flora are elucidated. This work aims to establish some concrete, complied, updated checklist of the endemic and near-endemic taxa in Egypt and presents an informative baseline for further cooperation toward conservation reasons. For conservationists, the distribution pattern of taxa within genera and families is proper (Fenner et al. 1997).

## Materials and methods

### Definitions of endemics and near-endemics

In this investigation, taxa occurring within the political borders of Egypt are referred to as” endemic,” and those known globally outside the Egyptian borders are referred to as” near-endemic.” As there is no perfect definition of” near-endemic,” we attempted to be as objective as achievable concerning the above-selected criterion.

### Data collection and data sources

For the compilation of this checklist, several data sources were used, such as monographs, articles, relevant floras (Al-Eisawi 1982 for Jordan; Gawhari et al. 2018 for Libya, Flora of Israel online, https://flora.org.il/en/plants; flora of Saudi Arabia online, http://plantdiversityofsaudiarabia.info/Biodiversity-Saudi-Arabia/Flora/Flora.htm; and flora of Lebanon online, http://lebanon-flora.org), and all recently revised taxonomic treatments of certain taxa. The main electronic sources and online global databases were used are: Global Biodiversity Information Facility (GBIF; http://www.gbif.org/occurrence), Plants of the World Online (POWO; http://www.plantsoftheworldonline.org), World Checklist of Selected Plant Families (WCSP; http://wcsp.science.kew.org/home.do), Euro + Med Plant Base (http://ww2.bgbm.org/EuroPlusMed/query.asp), African Plant Database (APD; http://www.ville-ge.ch/musinfo/bd/cjb/africa), International Plant Names Index (IPNI; http://www.ipni.org), World Flora Online (WFO; https://www.worldfloraonline.org), WFO Plant List (https://wfoplantlist.org/plant-list).

In addition, specimens kept in some local herbaria (Cairo University herbarium, CAI; Tanta University, TANE; Agricultural Museum, CAIM; Alexandria University, ALEX; Ain shams University, CAIA) were used, of which CAI is the richest specimen of endemic taxa of Egypt. These herbaria are part of the National Network of Egyptian Herbaria project (FLORA EGYPT), funded by the Egyptian Academy of Science and Technology, which aims to provide a database of Egyptian flora through specimens lodged in all local herbaria. The nomenclature of plant taxa was critically checked using (IPNI and POWO). Plant family circumscription follows the Angiosperm Phylogeny Group (APG III 2009) and Stevens (2001 onwards). Herbarium abbreviations are followed (Thiers 2023). This study used a combination of herbarium specimens and various relevant references to ensure that the scope and precision of the distribution data were reliable. Moreover, an effort was made to collect up-to-date distribution data to ensure the results reflect current distribution patterns. Based on the information we collected, we compiled a database of Egyptian endemic flora, including species names, family names, growth forms, habitats, distribution ranges at the phytogeographical region rank, and threat level of each species.

### Geographical distribution patterns, biological spectrum, and degree of differentiation

**Fig. 1:**
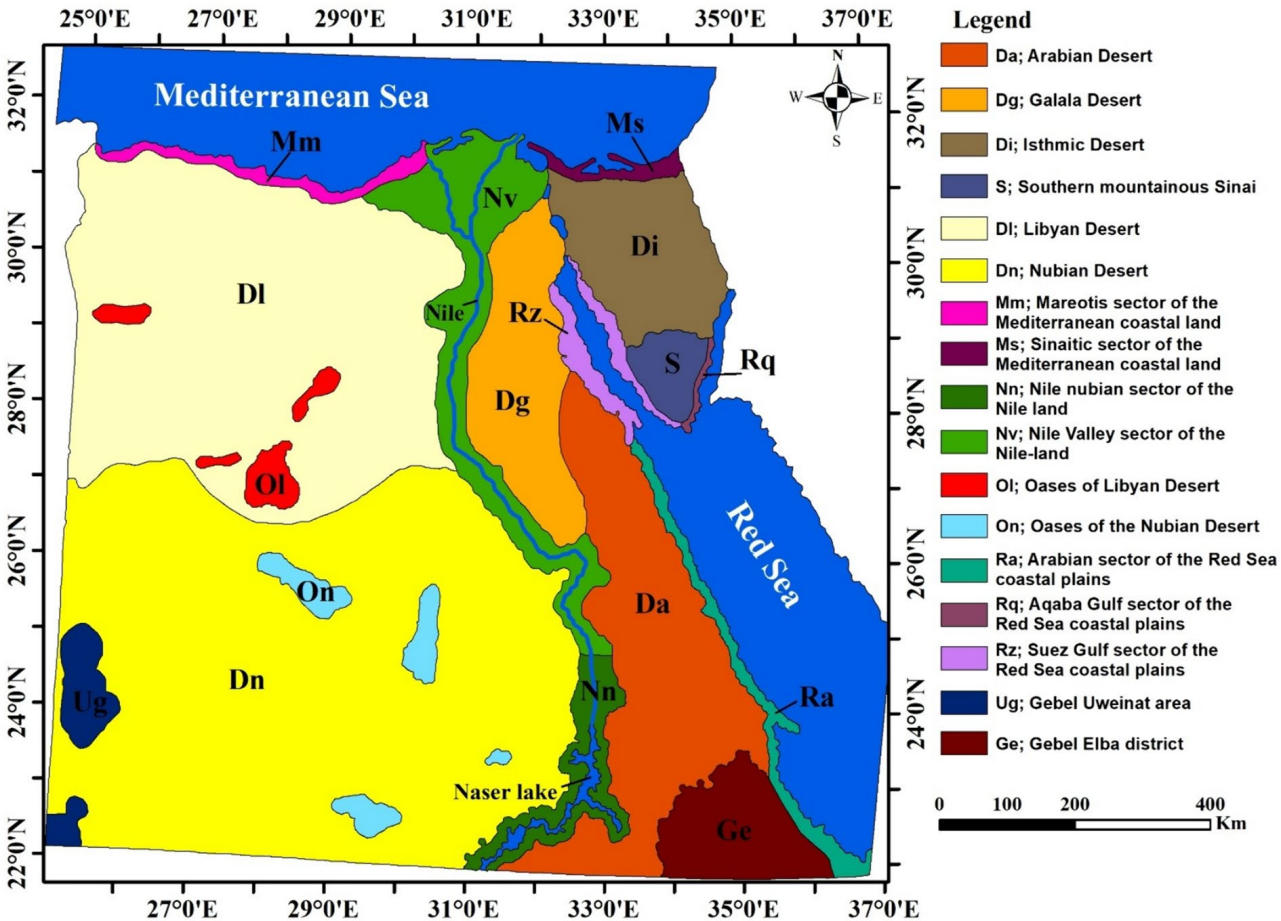
Phytogeographic regions (Operational Geographical Units, OGUs) of Egypt (after El Hadidi 2000)

The distribution patterns of endemic and near-endemic taxa among the different phytogeographical regions of Egypt (Fig. 1) were established based on the localities mentioned in the literature and on the labels of herbarium specimens. For each taxon, the distribution information was based on the existence or lack of each region’s endemic or non-endemic status. In the case of near-endemic taxa, the extent of the species' distribution outside of Egypt was documented by specifying the other nations or countries where the taxa are found. This study examined the distribution patterns of endemic and near-endemic taxa across the entire country.

Each phytogeographic region was referred to as an operational geographical unit (OGU) to detect the distribution patterns of endemic taxa. In this analysis, 14 OGUs were used (see Fig. 1 for full names). Distribution maps of endemic taxa were prepared using ArcGIS 10.4 software (ESRI 2016) and based on the geographical sites attained with GPS for each specimen within its OGU. Supplementary Tables 1 and 2 list Egypt’s endemic and near-endemic vascular plants (including infra-specific taxa), respectively.

For the analysis of the biological spectrum, the growth forms in both endemic and near-endemic species were classified into four types: trees (T), shrubs (S), perennial herbs (PH), and annuals (A). The t-test feature in SPSS version 16.0 for Windows assessed the significance of the growth form distribution patterns in each OGU.

Differentiation indices, which comprised a species differentiation index (Ds), a genus differentiation index (Dg), and a species–family differentiation index (Dsf), were used to describe the taxonomic degrees of divergence across endemic taxa (Huang et al. 2016).

The three indices' respective functions are displayed as follows:

Species differentiation index (D_s_) = N_s_/N_g_; Genus differentiation index (D_g_) = N_g_/N_f;_ Species–family differentiation index (D_sf_) = N_s_/N_f_, the numbers Ns, Ng, and Nf represent the number of endemic taxa, genera, and families of endemic flora, respectively, within an OGU.

### Assessment of endemism and beta diversity

For each OGU, assessment of endemism was based on two different indices: (1) single-region endemic taxa (SRET), which is the number of taxa endemic to a single phytogeographic region (OGU), and (2) multiple-region endemic taxa (MRET), which is the number of taxa endemic to more than one phytogeographic region (OGU). Beta diversity among the 14 OGUs was analyzed using the Chao-Jaccard index; it is more suitable for evaluating the similarity of samples of various sizes with many rare taxa and considers unobserved shared taxa (Chao et al. 2005). For this purpose, the program Estimate S for Windows version 7.5 (Colwell 2005) was used.

### Habitat specificity

In addition, each endemic taxon was assigned to the most preferable habitat. For this purpose, seven main habitats were determined: sandy plains and wadis (SPW), rocky plains and mountains (RPM), arable lands (AL), moist ground and canal banks (MGCB), stony ground (SG), coastal sandy plains (CSP), and dry salt marshes (DSM).

### Conservation status

The IUCN (2012; www.iucnreflist.org) is the most significant source of information about species conservation worldwide. Besides, El Hadidi and Hosni (2000) and Hosni et al. (2013) are used as well. The conservation status of each taxon was evaluated using the categories proposed by this system. In this study, a scale of seven categories was used as follows: Critically Endangered (CR), Data Deficient (DD), Endangered (EN), Extinct (EX), Least Concern (LC), Near Threatened (NT), and Vulnerable (VU).

### Statistical analyses

A presence/absence data matrix of 70 endemic taxa and 14 OGUs was established to construct homogeneous groups of phytogeographical regions. The R Development Core Team (2016) conducted an R-software study using the R-studio (RStudio 2015) interface. The “circlize” software created a chord diagram (Zuguang et al. 2014). The correlation coefficients were acquired to determine the relationship between the two variables with the help of the “Corrplot” package (Soetewey 2022). Blue with a 1 indicates a strong positive correlation, while white with a 0 indicates no relationship between the two variables. A significant negative association is indicated by red with a − 1. The data was scaled and standardized using complete as a linkage method after employing the Jaccard distance to create a visual dendrogram. The Principal Component study (PCA) was developed to visualize the distance matrices (Kassambara and Mundt 2017) utilized in the PCA study using the “factoextra” and “ggplot2” packages in R software.

Using the Multi-Response Permutation Procedure (MRPP; PC-ORD version 5 for Windows) with the Sørensen (Bray–Curtis) distance measure on a matrix of 14 OGUs and 70 endemic taxa, two test statistics were computed, and significant differences between floristic groupings were examined. The T statistic calculated the differences between groups. High segregation is indicated by a significant negative T value (≤ − 9.0), the more negative the test statistic, the larger the differences in species across the groups. The within-group homogeneity was estimated via the chance-corrected A statistic. Higher values of the A statistic indicated a high degree of homogeneity, ranging from (0.0–1.0). When there are numerous species, A is typically less than 0.1. An analysis of the null hypothesis, which states no differences between groups, was conducted by a Monte Carlo permutation process with a thousand permutations.

The resulting groups will henceforth be named ‘floristic groups.’ For near-endemic taxa, the classification of a presence/absence data matrix of 181 taxa and ten countries (including Egypt) Following the use of the Jaccard distance to produce a visual dendrogram, the data was scaled and standardized using complete as a linkage method, using the “factoextra” package in R.

## Results

### Taxonomic diversity and degree of differentiation

#### Endemic taxa

**Fig. 2:**
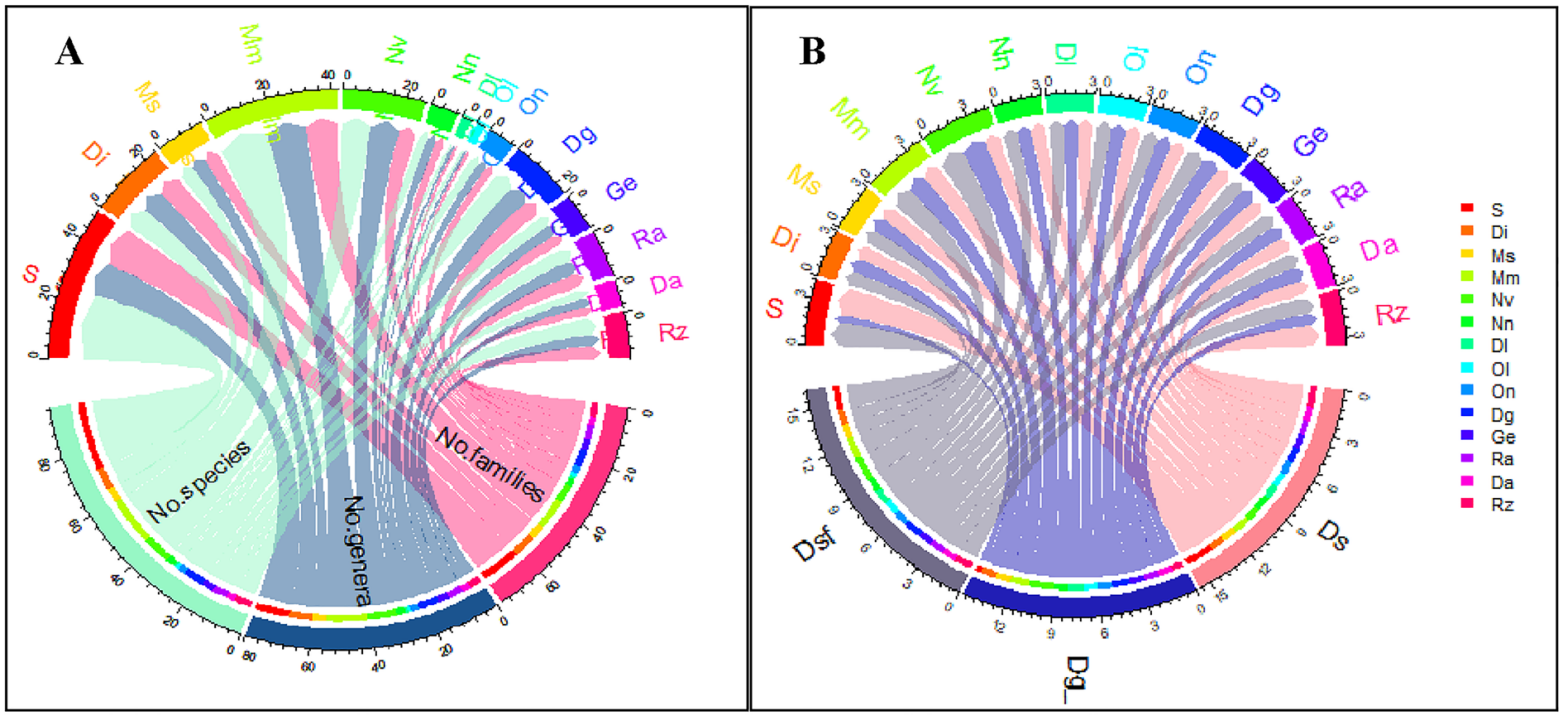
**A** Chord diagram for taxonomic diversity and **B** Degree of differentiation of endemic taxa in 14 OGUs. For abbreviations of OGUs, see Fig. 1, and for differentiation indices, see text

In total, 70 taxa (44 perennials, 26 annuals) of endemic plants belonging to 28 families and 56 genera were recognized (Supplementary Table 1), of which 19 taxa are new additions. Five families with the highest number of taxa: Asteraceae (9 taxa), Caryophyllaceae (8 taxa), Lamiaceae (7 taxa), Fabaceae (5 taxa), and Brassicaceae (4 taxa) constituted 47.1% of the total taxa. The OGUs with the highest numbers of families, genera and species were Sinai, Mm, Nv, and Di (Fig. 2A). Important genera with the highest number of taxa were *Silene* (6 taxa), *Euphorbia* (3 taxa), *Allium, Muscari, Anthemis, Ifloga, Teucrium, Limonium,* and *Fagonia* (2 taxa for each). The degree of differentiation among the endemic taxa was unevenly distributed across OGUs. Figure 2B showed that the species differentiation (D_s_) was the highest (2) in Sinai, the Suez Gulf sector of the Red Sea coastal plains (Rz) was 1.5, followed by the Mareotis sector of the Mediterranean coastal land (1.2). The remaining 11 OGUs were equally distributed. The genus differentiation (D_g_) was the highest (1.7) in the Nile Valley, followed by the Mareotis sector of the Mediterranean coastal land (Mm) with a value of 1.3, and the lowest (0.5) was in Sinai the species–family differentiation index (D_sf_) pattern followed the same pattern for genus differentiation.

#### Near-endemic taxa

One hundred and eighty-one near-endemic taxa (91 perennial herbs, 32 shrubs, 57 annuals and one tree) belonging to 39 families and 113 genera were recognized; of which 76 are new additions (Supplementary Table 2). For comparison, Table 1 showed similarities between the dominant endemic and near-endemic plant families, where Asteraceae (30 taxa), Fabaceae (17 taxa), Caryophyllaceae, and Lamiaceae (12 taxa for each) were the most species-rich families. The latter four families constituted 71 taxa (39.2%) of the total near-endemic taxa. Although Poaceae is the most representative family in the flora of Egypt (240 taxa; 11% of the total flora), it was not represented among the important families of the endemic taxa or in near-endemics. In addition to the previously reported endemic grass *Bromus aegyptiacus,* three grasses are newly added in this study, viz., *Bromus sinaicus* (Hack.) Tackh., *Eragrostis nitida* Link, and *Rostraria pumila* var. *glabrescens* (Täckh.) Hosni. Similarly, the near-endemic grasses included three newly added taxa, viz., *Aegilops longissima* Schweinf. & Muschl., *Trisetaria koelerioides* (Bornm. & Hack.) Melderis and *Stipagrostis shawii* (H. Scholz) H. Scholz (Supplementary Table 2). *Allium* (10 taxa), *Astragalus* and *Silene* (6 taxa for each), *Bellevalia* and *Centaurea* (5 taxa for each), and *Anthemis, Muscari, Verbascum,* and *Veronica* (4 taxa for each) included the highest numbers of species.

**Table 1:** Important plant families with the highest numbers and percentages (between parentheses) of endemic and near-endemic taxa compared to the most species-rich families for the total flora of Egypt (after Boulos 1995–2009)

Endemics	No of taxa (%)	Near-endemics	No of taxa (%)	Total flora	No of taxa (%)
Asteraceae	9 (12.8)	Asteraceae	30 (16.6)	Poaceae	240 (11.0)
Caryophyllaceae	8 (11.4)	Fabaceae	17 (9.4)	Fabaceae	235 (10.7)
Lamiaceae	7 (10.0)	Caryophyllaceae	12 (6.6)	Asteraceae	230 (10.4)
Fabaceae	5 (7.1)	Lamiaceae	12 (6.9)	Brassicaceae	110 (5.0)
Brassicaceae	4 (5.7)	Amaryllidaceae	11 (6.1)	Amaranthaceae	96 (4.4)
Amaryllidaceae	3 (4.3)	Asparagaceae	11 (6.1)	Caryophyllaceae	83 (3.8)
Asparagaceae	3 (4.3)	Brassicaceae	10 (5.5)	Lamiaceae	54 (2.4)
Euphorbiaceae	3 (4.3)	Apiaceae	7 (3.9)	Euphorbiaceae	53 (2.4)
Zygophyllaceae	3 (4.3)	Plantaginaceae	6 (3.3)	Apiaceae	51 (2.3)
Total taxa	70		181		2200

### Biological spectrum

#### Endemic taxa

**Fig. 3:**
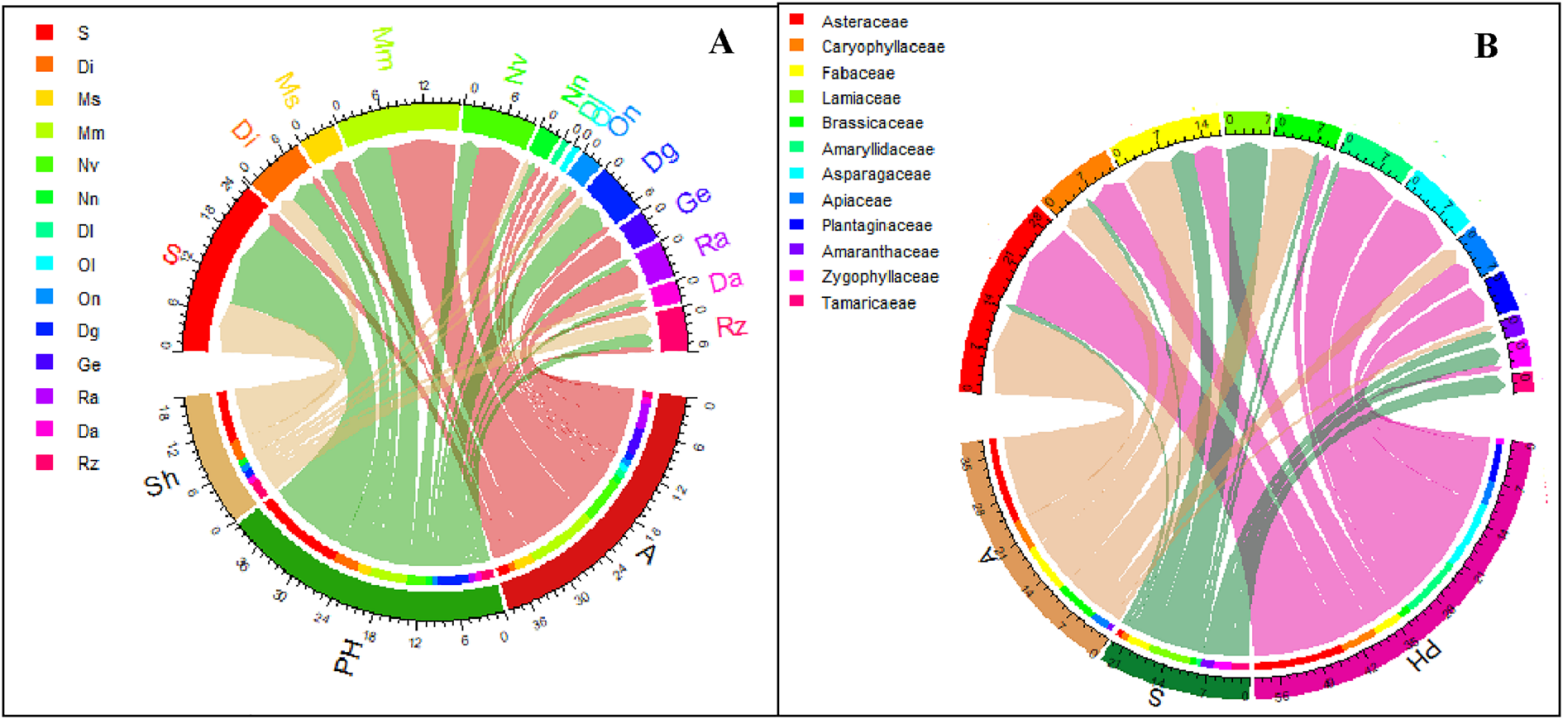
**A** Chord diagrams showing the distribution of growth forms of the endemic taxa in each of the 14 OGU, and **B** The distribution of growth forms in the most important families of near-endemic taxa. *S* shrubs, *PH* perennial herbs, *A* annuals. For abbreviations of OGUS, see Fig. 1

None of the endemic trees were represented among other growth forms (Supplementary Table 1). Regarding the total number of endemic taxa, shrubs, and perennial herbs had the highest number (44 taxa, 62.8%), and a lower proportion were for annuals (26 taxa, 37.2%). Asteraceae had the highest numbers of annuals (6 taxa), followed by Brassicaceae (5 taxa), Caryophyllaceae, and Fabaceae (3 taxa for each)**.** The chord diagram visualized the distribution of growth forms in each of the 14 OGUs (Fig. 3A). The highest numbers of shrubs (8 taxa, 72.2% of the total shrubs) and perennial herbs (14 taxa, 42.4% of the total perennial herbs) were recorded from the mountainous Sinai (S). The annuals showed their highest record in (Mm) with 11 taxa (42.2% of the total annuals), followed by (Nv) with seven taxa (27%). Annuals in the Dl, Ol, and GE regions represented the endemic taxaWhereas the distribution patterns of growth forms in each OGU showed insignificant differences (results not shown) for Nn (*p* = 0.08), Dl (*p* = 0.31), Ol (*p* = 0.31), On (*p* = 0.08) and Da (*p* = 0.08), the distribution patterns of the remaining OGUs differed significantly (*p* ranged between 0.001 and 0.01).

#### Near-endemic taxa

The general distribution of the 181 near-endemic taxa in the different categories indicated the dominance of the perennial herbs (91 taxa, 50%), followed by annuals (57 taxa, 31%). The tree was the least represented (one taxon, 1%), whereas *Medemia argun* was the only recorded taxon. Families with the highest number of perennial herbs were Asteraceae (15 taxa), Asparagaceae (11 taxa), Amaryllidaceae (10 taxa), Caryophyllaceae and Plantaginaceae (6 taxa for each), and Fabaceae and Lamiaceae (5 taxa for each). Thirty-two shrubs (18%) were enumerated from 17 families, of which Lamiaceae, Fabaceae, Tamaricaceae, and Zygophyllaceae included the highest numbers of taxa (7, 4, 3 and 3, respectively) (Fig. 3B). Amongst other taxa, *Verbascum letourneuxii, Origanum isthmicum*, *Pterocephalus sanctus*, *Taverniera aegyptiaca* and *Zygophyllum dumosum* can be mentioned. Perennial herbs and shrubs constituted about 68% of the total near-endemics. Annuals constituted 57 taxa (31%) of the total near endemics belonging to 16 families, where Asteraceae (14), Fabaceae (8), Brassicaceae (7), and Caryophyllaceae (5) included the highest numbers of taxa. Such annuals included, amongst others, *Centaurea glomerata*, *Senecio glaucus* subsp. *glaucus, Crepis aculeata, Lotus nubicus,* and *Silene palaestina*.

### Geographical distribution patterns and beta diversity

#### Endemic taxa

**Fig. 4:**
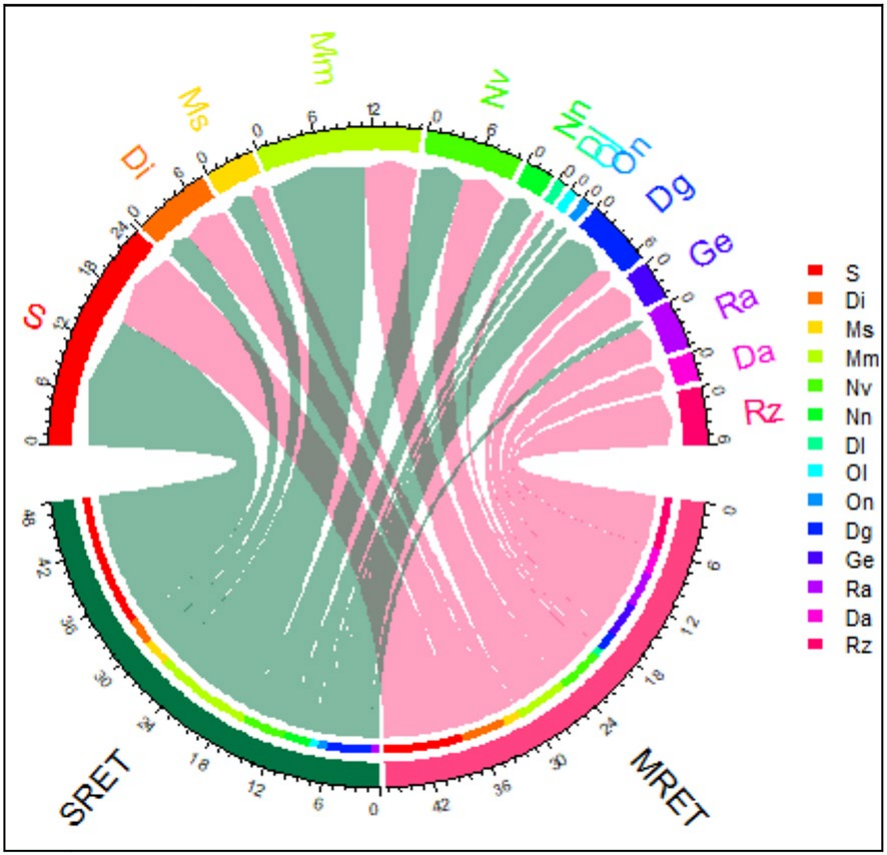
Chord-diagram for geographical distribution patterns of endemic taxa: multiple region (MRET), and single region (SRET) in each of the 14 OGUs

Based on their geographical distribution, two patterns were identified: (1) multiple region endemic taxa (MRET) that are widely distributed and recorded in more than one OGU, and (2) single region endemic taxa (SRET) that are narrowly distributed and recorded in one OGU. As revealed from analysis, 20 taxa (about 28.6% of the endemic taxa) were identified as multiple region endemic taxa (MRET). Among the OGUs, the mountainous Sinai (S) included the highest number of endemic taxa (24 taxa, 34.3% of the total endemics), followed by the Mareotis sector of the Mediterranean coastal land (Mm) with 17 taxa (24.3%) and the Nile Valley (Nv) with ten taxa (14.3%; Fig. 4).

The lowest numbers of endemic taxa were found in the Libyan Desert (Dl) and Oases of the Libyan Desert (Ol), both with single taxons (1.4%). *Hyoscyamus boveanum* occurred in 5 of the 14 OGUs (Supplementary Fig. 1A), mainly distributed in the Arabian Desert (Da), along the Suez Gulf of the Red Sea (Rz), the Isthmic Desert (Di), the mountainous Sinai (S), and the Nile Valley (Nv). Sixteen taxa (80%) were found in two OGUs, none of which was known from the Libyan Desert (Dl) and its Oases (Ol and On): *Nasturtiopsis integrifolia* (Brassicaceae), *Euphorbia obovata* (Euphorbiaceae), and *Teucrium leucocladum* var. *glandulosum* (Lamiacaeae). On the other hand, 4 taxa were confined to the OGUs located east of the country (Dg, Ge, Ra, Da and Rz), such as *Ifloga spicata* subsp. *elbaensis* (Asteraceae), *Biscutella didyma* var. *elbensis* (Brassicaceae), *Silene villosa* var. *erecta* (Caryophyllaceae) and *Solanum nigrum* var. *elbaensis* (Solanaceae). Two endemic species, *Pancratium arabicus* (Amaryllidaceae) and *Euphorbia punctata* (Euphorbiaceae), were confined to the Mediterranean regions (Ms and Mm). *Euphorbia obovata* (Euphorbiaceae) and *Origanum syriacum* subsp. *sinaicum* (Lamiaceae) were confined to the OGUs of the Sinai Peninsula (Di and S).

The single region endemic taxa (SRET; Supplementary Fig. 1B) comprised 50 taxa (71.4%), with the highest proportions from the mountainous Sinai OGU (15 taxa, 30%) and the Mediterranean OGUs (14 taxa). In terms of characteristic endemic taxa, *Bufonia multiceps, Rosa arabica*, *Primula boveana*, and *Euphorbia sanctae-catharinae* characterized the Sinai (S) OGU (Supplementary Fig. 1C), whereas *Allium mareoticum, Muscari albiflorum, Veronica anagalloides* subsp. *taeckholmiorum,* and *Limonium mareoticum* characterized the (Mm) OGU. The lower proportion (4 taxa) was counted from the oases of the Libyan Desert, such as *Ducrosia ismaelis*, *Melilotus serratifolius*, *Rhazya greissii,* and *Apium graveolens* var. *bashmensis.* Remarkably, none of the SRET groups were confined to the Gebel Elba region (Ge) and Libyan Desert region (Dl).

Pearson’s correlation coefficients between different pairs of aspects of endemism (Table 2) indicated that the genus differentiation index (Dg) was insignificantly negatively correlated with other aspects. In contrast, the species-family differentiation index was positively correlated with most of the measured aspects. MRET and SRET showed significant positive correlations with each other (*r* = 0.63) and with most of the examined aspects.

**Table 2:** Pearson’s correlation coefficients (*r*) between aspects of endemism

Aspects of endemism	No families	No genera	No species	Ds	Dg	Dsf	MRET
No families							
No genera	0.93^a^						
No species	0.97^a^	0.91^a^					
Ds	0.74^a^	0.47	0.77^a^				
Dg	− 0.16	0.22	− 0.14	− 0.61^b^			
Dsf	0.74^b^	0.82^a^	0.85^a^	0.62^b^	0.23		
MRET	0.85^a^	0.76^a^	0.83^a^	0.74^a^	− 0.18	0.71^a^	
SRET	0.90^a^	0.87^a^	0.95^a^	0.68^a^	− 0.09	0.81^a^	0.63^b^

*Ds* Species differentiation index, *Dg* Genus differentiation index, *Dsf* Species–family differentiation index, *MRETS* Multiple region endemic taxa, *SRET* Single region endemic taxa

^a^Correlation is significant at the 0.01 level

^b^Correlation is significant at the 0.05 level

For estimation of beta-diversity, the Chao-Jaccard index (C-J; Supplementary Table 3A) showed a clear separation between the endemic taxa in the 14 OGUs with high similarity between Di and both S (C-J = 0.7) and Rz (C-J = 0.8). Similar high similarities (C-J = 0.9) were found between Mm and both Nv and Dl. As expected, the endemic taxa in Gebel Elba (Ge) showed high similarity with Ra (C-J = 0.9). The highest numbers of shared taxa (7 taxa) occurred between S and Rz, between Mm and Nv (5 taxa), and between Ge and Ra (5 taxa).

Sixty taxa showed occurrences in two OGUs, where 24 taxa (40% of this group) were mainly recorded from the Mediterranean OGUs (Mm and Ms) and penetrated the Sinai OGUs (Di and S), such as *Allium curtum* subsp*. palaestinum, Bellevalia eigii, Alkanna strigosa, Trigonella arabica,* and *Lycium schweinfurthii* var. *aschersohnii.* Another 25 taxa (41.7% of this group) showed penetration of their distribution from the northern OGU (Di) toward the southern OGU (S) of the Sinai Peninsula, including *Ferula sinaica, Eremogone sinaica, Origanum isthmicum, Convolvulus palaestinus,* and *Teucrium jordanicum* var. *jordanicum*.

#### Near-endemic taxa in Egypt

The similarity between the recorded near-endemic taxa in previous works (Boulos 2009; Hosni et al. 2013; Shaltout et al. 2018, POWO, and this study using Jaccard’s index (Supplementary Table 3B).) indicated low similarity indices between taxa of this study and other works, but higher similarity index (0.89) with the taxa identified by POWO. Studies by Hosni et al. (2013) and Shaltout et al. (2018) showed high similarity indices with that of Boulos (2009).

The analysis of the occurrences of the 181 near-endemic taxa in studied OGUs revealed that *Reseda pruinosa* and *Allium tel-avivense* (Supplementary Table 4) had a wide range of distributions as found in more than four OGUs, mainly along the Mediterranean coast (Mm and Ms), in the Sinai Peninsula (Di and S), along the Red Sea coast (Ra and Rz), and in the Eastern Desert (Dg and Da).

Seventy-nine taxa had a narrow range of distribution (confined to one OGU); the highest number (43 taxa, 44% of this group) were known from Sinai OGUs (Di and S) such as *Saltia papposa, Astragalus amalecitanus, Pterocephalus sanctus, Veronica kaiseri, Zygophyllum propinquum* subsp*. migahidii, Lupinus palaestinus*, and *Silene hussonii,* followed by 19 taxa (25.3% of this group) from the Mediterranean region (Mm and Ms., OGUs) such as *Crepis aculeata, Nigella arvensis* subsp. *negevensis*, *Bupleurum nanum, Herniaria cyrenaica*, and *Ebenus armitagei*.

#### Habitat specificity of endemic taxa

None of the endemic taxa were represented in all identified habitats. Two major habitats harboured the highest numbers of endemics: the sandy plains and wadis (SPW, 24 taxa) and the rocky plains and mountains (RPM, 19 taxa), which together constituted more than 60% of the taxa (Supplementary Table 5). The coastal sandy plains (CSP) and the dry salt marshes (DSM) included the lowest numbers of taxa; two for the former and one for the latter. Compared with other OGUs, the mountainous Sinai (S) included the highest taxa in the SPW, RPM, and SG. Similar trends can be seen in AL, where the highest taxa occurred in Nv.

Some taxa showed consistency to a certain habitat such as *Hyoscyamus boveanum* and *Pancratium arabicum* in sandy plains and wadis, *Allium crameri* and *Pterocephalus arabicus* in rocky plains and mountains, *Bromus aegyptiacus* and *Scorzonera drarii* in arable lands, *Sonchus macrocarpus* and *Apium graveolens* var. *bashmensis* in moist ground and canal banks, *Origanum syriacum* subsp. *sinaicum* and *Ballota kaiseri* in stony ground, *Atractylis carduus* var. *marmarica* in coastal sandy plains, and *Limonium mareoticum* in dry salt marshes). Shrubs and perennial herbs dominated both rocky plains and mountains (RPM) and stony ground (SG), but annuals dominated the arable land (AL) habitat.

#### Conservation status of endemic taxa

In general, an assessment of conservation based on IUCN categories revealed that four major categories (CR, EN, EX, and VU) constituted 59 taxa (84.3% of endemics); the highest proportion was for critically endangered (26 taxa, 37%), followed by equal contribution (11 taxa) of the remaining three categories.

**Fig. 5:**
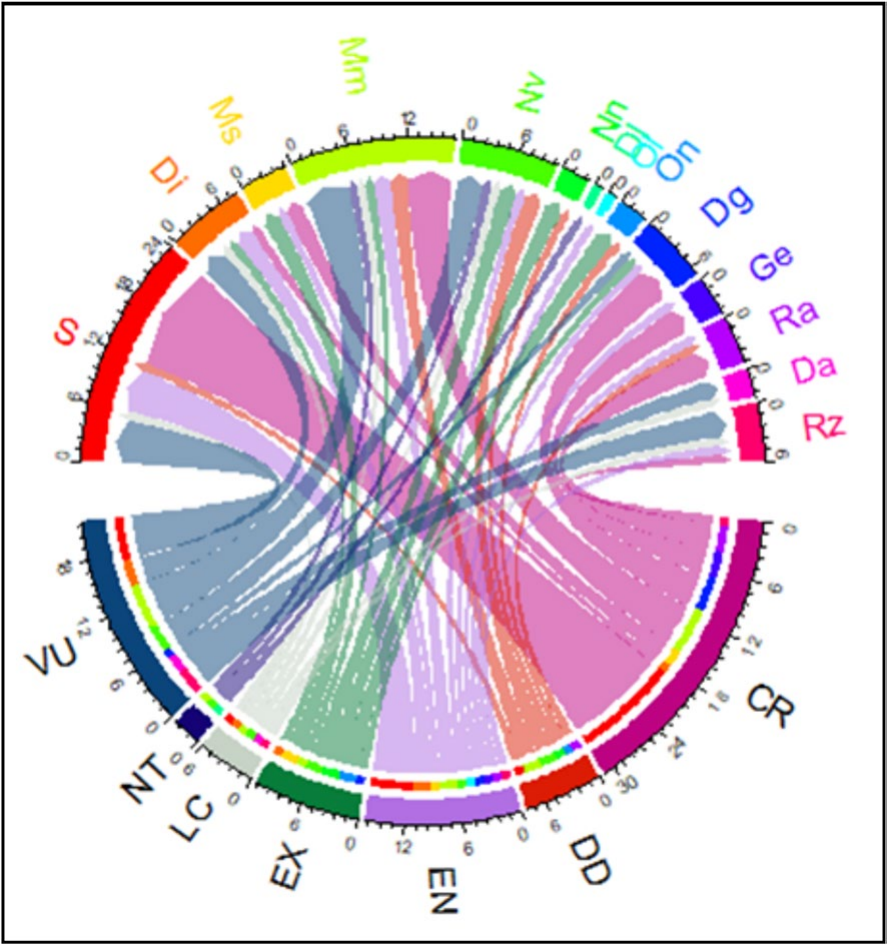
Distribution of IUCN categories of the numbers of endemic taxa in each OGU, IUCN Abbreviations: *CR* critically endangered, *VU* vulnerable, *EN* endangered, *EX* extinct, *DD* data deficient, *LC* least concern, *NE* near threatened

The endemic taxa distribution across IUCN categories in various habitats showed that 17 (54.8%) out of the 31were critically endangered taxa and 12 (54.5%) out of 22 were vulnerable taxa that recorded from (S) and (Mm). The near threatened (NT) and least concern (LC) categories had the fewest taxa: three for NT and six for LC, with one taxon shared by Mm and Nv. In contrast, the mountainous Sinai (S) had the highest number of taxa in the critically endangered (CR), endangered (EN), and vulnerable (VU) categories, with twelve, five, and five taxa, respectively. Meanwhile, the Mareotis sector of the Mediterranean coastal region (Mm) has five critically endangered and vulnerable taxa (Fig. 5).

Among the 17 families (34%) that represent the critically endangered taxa, Caryophyllaceae and Lamiaceae have the highest numbers of taxa (5 for the former and 3 for the latter). While Asteraceae and Fabaceae had the highest numbers in the extinct (EX) taxa, Asteraceae, Brassicaceae, and Lamiaceae were best represented in the vulnerable taxa (2 for each). *Silene* and *Euphorbia* were highly represented in the CR, whereas *Teucrium* was best represented in the VU taxa.

#### Correlation between habitat specificity and conservation status of endemic taxa

**Fig. 6:**
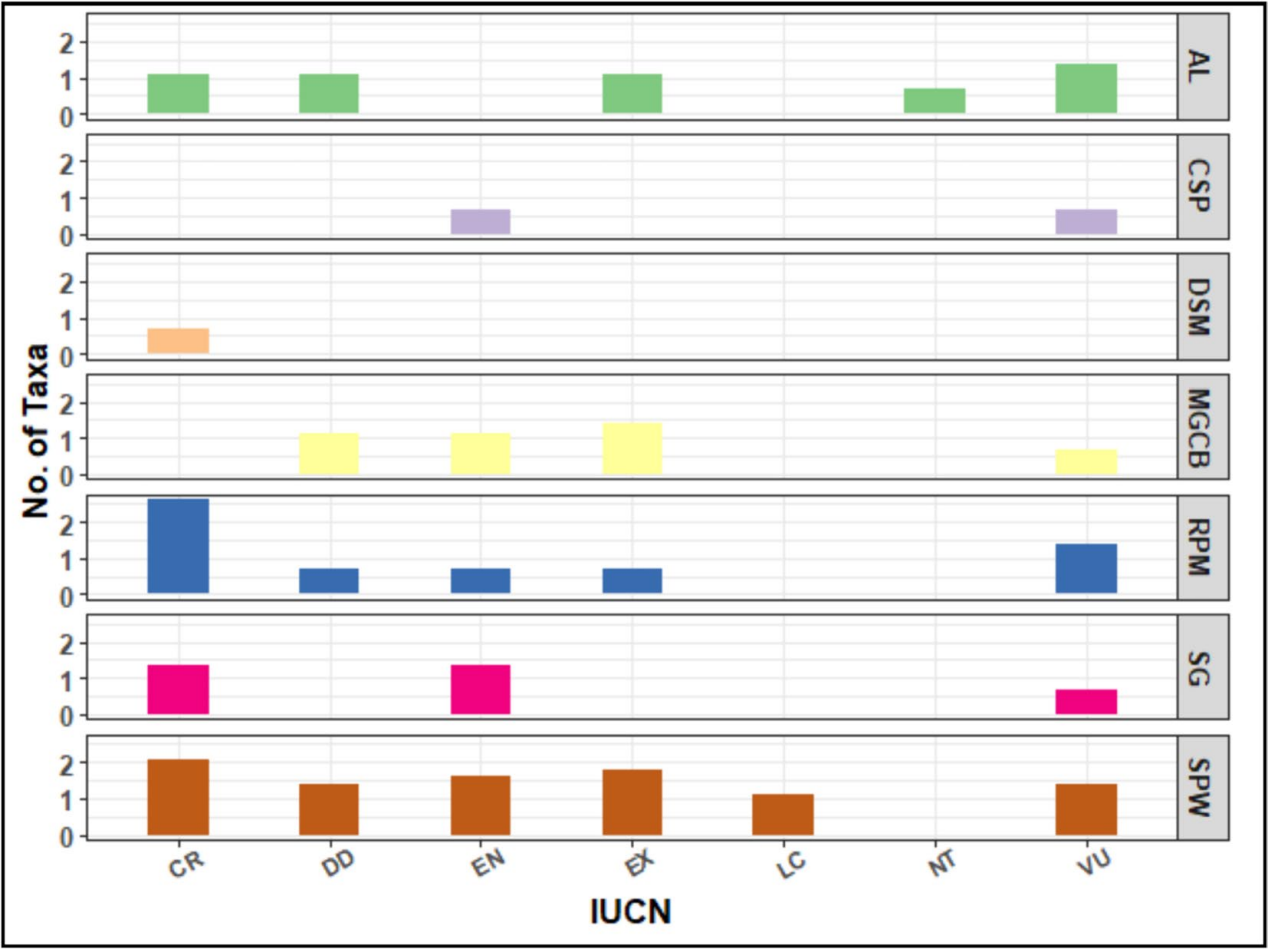
Endemic taxa distribution across IUCN categories in various habitats. Abbreviations of IUCN categories: *CR* critically endangered, *VU* vulnerable, *EN* endangered, *EX* extinct, *DD* data deficient, *LC* least concern, *NT* near threatened, Habitats’ abbreviations: *AL* arable lands, *CSP* coastal sandy plains, *DSM* dry salt marshes, *MDCB* moist ground and canal banks, *RPM* rocky plains and mountains, *SG* stony ground (SG), *SPW* sandy plains and wadis. For abbreviations of OGUs, see Fig. 1

While the sandy plains and wadis (SPW, 24 taxa) and the rocky plains and mountains (RPM, 19 species) included most endemic taxa (43 taxa, 61.4%), the coastal sandy plains (CSP) and the dry salt marshes (DSM) were the least represented. The taxa inhabited RPM and SPW (Fig. 6) constituted the bulk of the critically endangered taxa (CR): 13 for the former and 7 for the latter. Meanwhile, the highest numbers of taxa in the EN and EX categories were from the sandy plains and wadis (SPW).

**Fig. 7:**
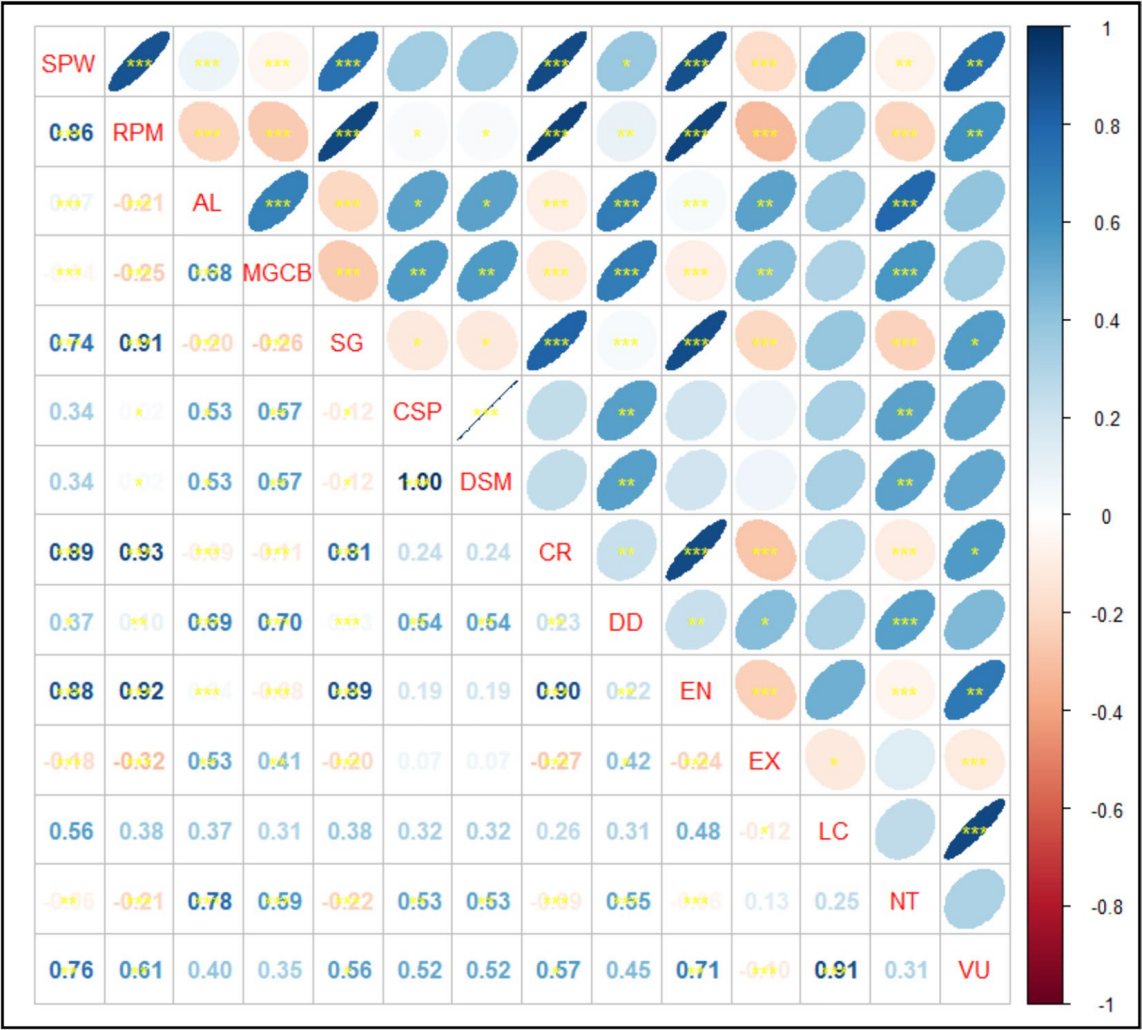
Pearson’s correlation coefficients (*r*) between habitats and IUCN categories of endemic taxa. Abbreviations of habitats: *SPW* sandy plains and wadis, *RPM* rocky plains and mountains, *AL* arable lands, *MGCB* moist ground and canal banks, *SG* stony ground, *CSP* coastal sandy plains, *DSM* dry salt marshes. For abbreviations for IUCN categories, see Table 6. *** = correlations are significant at the 0.001 level, ** = correlations are significant at the 0.01 level, * = correlations are significant at the 0.05 level

In terms of numbers of taxa, the pairwise correlations between different types of habitats of the endemic taxa (Fig. 7)., the taxa of sandy plains and wadis (SPW), and the rocky plains and mountains (RPM) were strongly correlated with each other (*r* = 0.86) and with the stony ground habitat (SG, *r* = 0.74 for the former and *r* = 0.91 for the latter). The moist ground and canal banks (MGCB) showed high correlations with those of the arable lands (AL, *r* = 0.68). A strong correlation occurred between the taxa of the coastal sandy plains and those of the dry salt marshes (DSM, *r* = 0.94). To examine the differences between habitats of the endemic taxa and their occurrences in OGUs, the results of the t-test revealed that Di and Mm were significantly different (*p* = 0.047 for the former and *p* = 0.009 for the latter).

Results of the ANOVA test indicated highly significant variations (F = 12.45, *p* = 0.001) between categories of IUCN. Although Pearson’s correlation between the different types of habitats and IUCN categories (Fig. 7) showed a negative non-significant relation (*r* = − 0.120, *p* = 0.33), the correlation between aspects of endemism and IUCN categories was highly negatively significant (*r* = − 0.39, *p* = 0.001).

The application of stepwise multiple regression analysis using the four variables: type of endemism (SRET and MRET), growth forms (GF), IUCN categories, and habitats (Table 3) indicated that the types of endemism and IUCN categories had the highest values of R^2^ (0.159 and 0.173, respectively) with significantly higher variations.

**Table 3:** Results of stepwise multiple regression analysis for 4 dependent variables of the 70 endemic taxa in 14 OGUs

Dependent variable	Multiple R	R^2^	Adjusted R^2^	F	p	Intercept ± S.E	Beta values (coefficients)		
							E	H	IUCN
Endemism type (E)	0.399	0.159	0.121	4.164	0.009^a^	2.038 ± 0.19			
Habitats (H)	0.318	0.101	0.060	2.481	0.068	4.565 ± 1.32	− 0.07		
IUCN categories	0.416	0.173	0.135	4.594	0.005^a^	6.702 ± 1.21	0.40^a^	0.15	
Growth forms (GF)	0.290	0.084	0.042	2.021	0.119	1.182 ± 0.48	0.012	0.28^b^	0.54

*S.E.* Standard error

^a^Significant at the 0.01 level

^b^Significant at the 0.05 level

### Floristic regionalization

#### Endemic taxa

**Fig. 8:**
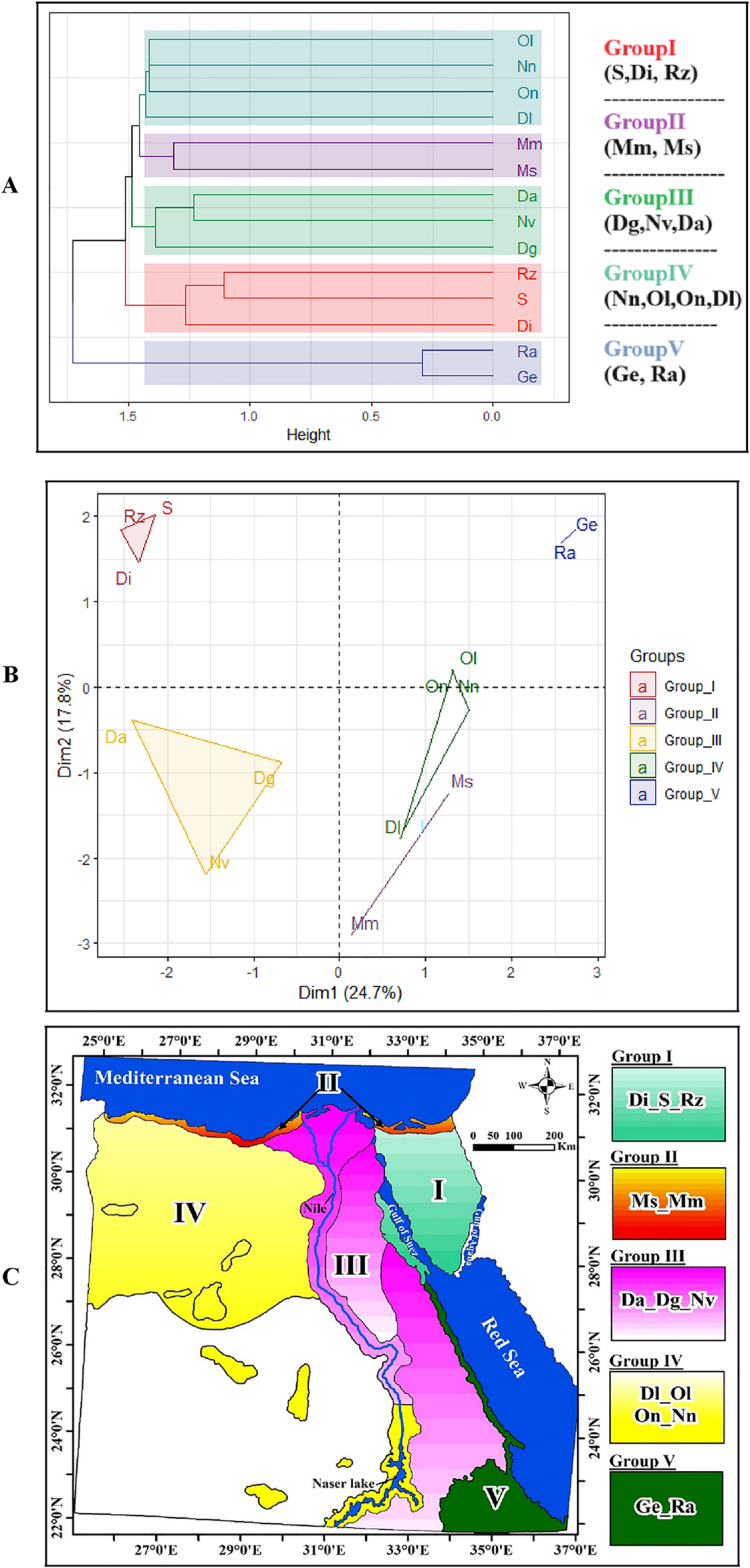
**A** Cluster dendrogram, **B** PCA of the 14 OGUs using Jaccard’s index as a distance measure indicating the separation of the five floristic groups (I-V) of endemic taxa, and **C** Distribution of floristic groups obtained from cluster analysis. For abbreviations of OGUs, see Fig. 1

Cluster analysis of endemic taxa and principal components analysis (PCA) revealed the presence of distinct groupings (Fig. 8A). PCA further supported these groupings (Fig. 8B). This led to the projection of floristic groups (I–V) onto the 14 studied OGUs (Fig. 8C). The resulting dendrogram formed two clusters (Fig. 11B): the larger encompassed four subclusters (4 Groups), while the smaller consisted of the fifth group. Notably, the first four dimensions accounted for 63.96% of the total variation, with the first two dimensions representing 42.45% of the variation.

**Group (I)** occupies the first subcluster of the dendrogram (Fig. 8A) and the easternmost OGUs of Egypt (Sinai Proper, Isthmic Desert, and the Suez Gulf sector of the Red Sea coastal plains), which are geographically very close together (Fig. 8B). This group included 28 taxa; the majority (24 taxa) was recorded from (S). The two taxa *Polygala sinaica* var. *sinaica*, *Hyoscyamus boveanus* shared the three OGUs. Four taxa showed consistency to (Di): *Allium crameri, Scorzonera drarii*, *Brassica deserti,* and *Zygophyllum migahidii* Hadidi var. *isthmia*.

**Group (II)** included OGUs in both sectors of the Mediterranean coastal land, representing the country's northern borderline. This group included 20 taxa, of which 17 were exclusively found in (Mm), such as *Bassia aegyptiaca*, *Anthemis microsperma*, *Veronica anagalloides* subsp: taeckholmiorum*, Silene apetala* var*. glabrata,* and *Bromus aegyptiacus*. Two taxa, *Euphorbia punctata* and *Pancratium arabicum* were shared between the two OGUs of the Mediterranean region (Mm and Ms).

**Group (III)** is located in the middle of the dendrogram and includes three OGUs, two representing the North–South stretches of the Eastern Desert (Da and Dg) and the adjacent Nile Valley sector of Nile land. Seventeen taxa were included in this group, of which *Sonchus macrocarpus* was the only common among the three OGUs of this group. The (Nv) hosts the highest number of taxa (10), while (Da) is the least (3). Whereas ten taxa were common between (Nv) and (Da), specific taxa were exclusively recorded from the (Nv), such as *Sinapis arvensis* subsp. *allionii*, *Fagonia taeckholmiana*, *Persicaria obtussifolia,* and *Trigonella media*.

**Group (IV), **apart from the Nubian Nile (Nn), included the three OGUs of the Western Desert. None of the eight taxa included in this group were common between the 4 OGUs (Nn, Dl, Ol, and On). Among the characteristic taxa, *Apium graveolens* var. *bashmensis* (Ol)*, Atriplex nilotica* (Nn), *Anthemis retusa* (Dl), *Melilotus serratifolius,* and *Rhazya greissii* (On) were recorded.

**Group (V)** included five taxa from two floristically and geographically related OGUs (Ra and Ge) and was placed in the lower part of the dendrogram. Apart from *Dicliptera aegyptiaca* that showed consistency to the (Ra), the remaining four taxa were common: *Biscutella* *didyma* var. *elbensis*, *Ifloga spicata* subsp. *albescens, Silene villosa* var. *erecta* and *Solanum nigrum* var. *elbansis*.

The Multi-Response Permutation Procedures (MRPP) test revealed certain significant differences between floristic groups in the floristic matrix (chance-corrected within-group agreement *A* = 0.17; p < 0.0009), suggesting separated assemblages (Table 4). The *T* statistic was − 9.20, indicating dissimilarity among the 14 studied OGUs. The pairwise comparisons showed significant differences between most of the groups. Floristic differences between groups (II) and (III), between groups (II) and (V), and between (III) and (V) were insignificant. The five groups (I –V) occupied different positions in the species space, as shown by the strong chance-corrected within-group agreement (*A*) and test statistics (*T*).

**Table 4:** Multiple pairwise comparisons of the MRPP statistics of the floristic groups (I-V) based on Bray–Curtis distance

Group compared	*T*	*A*	*P* value
I vs. II	− 2.13	0.092	0.00000000^a^
I vs. III	− 2.58	0.062	0.024^b^
I vs. IV	− 3.53	0.055	0.009^b^
I vs. V	− 2.23	0.34	0.00000000^b^
II vs. III	− 1.47	0.041	NS
II vs. IV	− 2.49	0.023	0.02^b^
II vs. V	− 1.41	0.35	NS
III vs. IV	− 2.68	0.029	0.01^b^
III vs. V	− 2.22	0.311	NS
IV vs. V	− 3.00	0.22	0.02^b^

*A* change-corrected within group agreement, *T* difference between the observed and expected deltas

^a^Significant level at 0.01

^b^Significant level at 0.05, NS = Not significant

#### Near-endemic taxa

Concerning the number of taxa extended to a country (countries) outside Egypt, Libya was the highest as extended in one country, followed by Palestine. In contrast, Palestine and Jordan were the highest as extended in two countries, and Palestine, Jordan, and Lebanon-Syria were the highest in three countries (Supplementary Fig. 2).

**Fig. 9:**
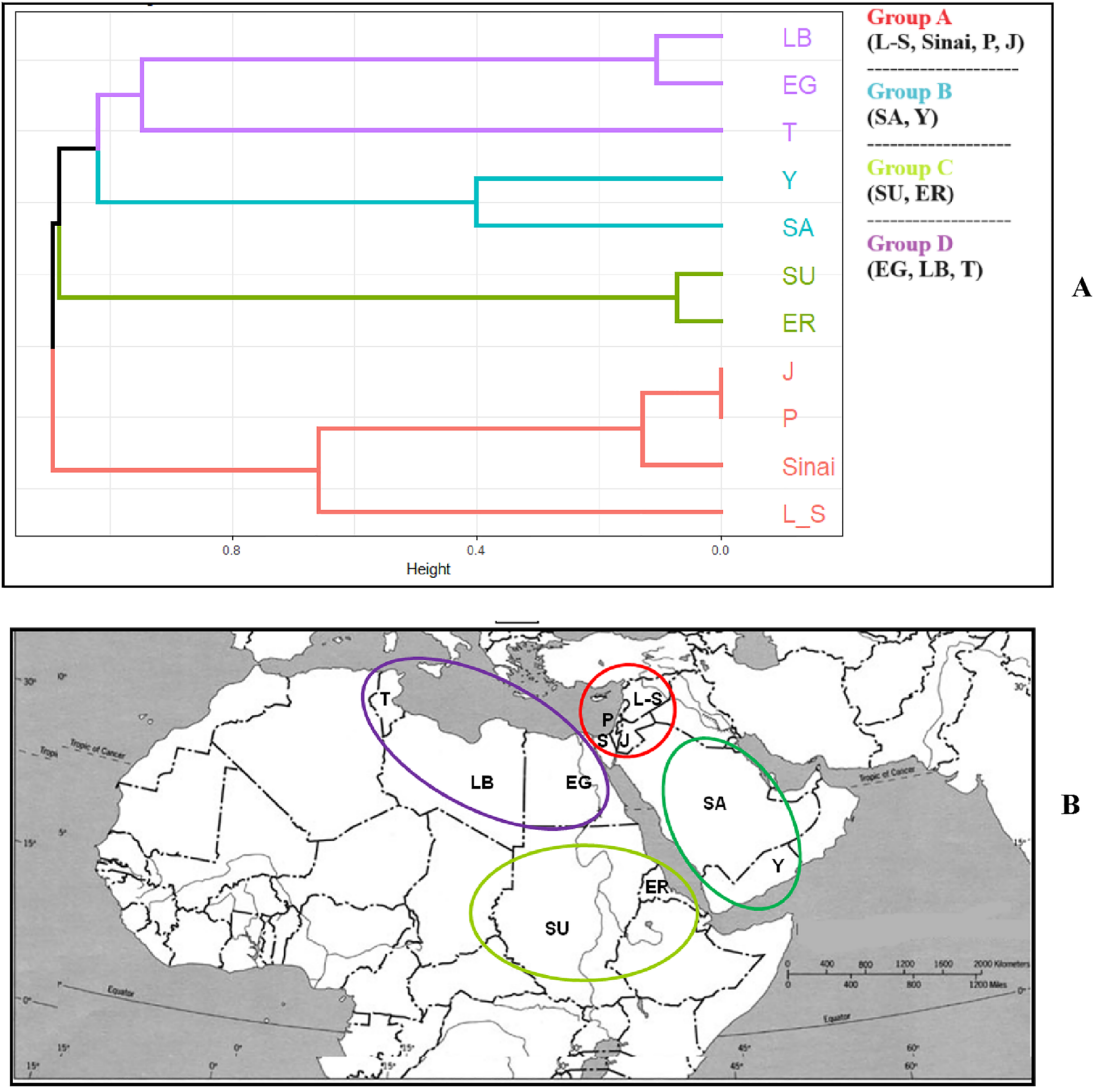
**A** UPGMA cluster analysis of 181 near-endemics distributed in 10 countries, **B** Distribution patterns of the near-endemic taxa in adjacent countries. EG, *S* Egypt and Sinai, *LB* Libya, *T* Tunisia, *SU* Sudan, *ER* Eritrea, *P* Palestine, *L-S* Lebanon-Syria, *J* = Jordan, *SA* Saudi Arabia, *Y* Yemen

After applying UPGMA cluster analysis on 181 near-endemic taxa in 10 countries using the Jaccard method, four main cluster groups (A–D) were obtained (Fig. 9A). Group (A) was the North-eastern-Mediterranean-group represented by Sinai, Palestine, Jordan, Lebanon, and Syria; Group (B) was the Saharo-Arabian-Asiatic group represented by Saudi Arabia and Yemen; Group (C) was the Sudanian-East African group represented by Sudan and Eritrea, and group (D) was the Mediterranean-North African group represented by Egypt, Libya, and Tunisia. When locating the position of these groups on maps of the adjacent countries (Fig. 9B), overall distribution patterns can be detected and described as follows:

I. North African extension: This group included 32 taxa (15 annuals, 11 perennial herbs, six shrubs) of Mediterranean origin recorded along the northwestern Mediterranean coast of Egypt. Twenty-six taxa occurred in Egypt and Libya, and four taxa were found in Libya and Tunisia other than Egypt. *Crepis libyca, Nonea vivianii, Herniaria cyrenaica, Silene fruticosa* subsp*. cyrenaica*, *Ebenus armitagei*, *Bellevalia sessiliflora*, and *Nigella arvensis* subsp. *taubertii* were examples of this group.
II. Sudanian extension: This group comprises seven taxa, mostly of perennial herbs. Outside Egypt, five taxa extended their distribution into Sudan, and the other two into Sudan and Eritrea. These taxa occurred in south and southeast Egypt and penetrated north and northeast Sudan. The endangered desert tree *Medemia argun* and two taxa of aquatic habitat (*Veronica anagallis-aquatica* var. *nilotica* and *V. scardica* subsp. *africana*) were examples of this group.
III. East Mediterranean extension: This group constituted 93 taxa (53.6% of the total near-endemics), of which 60 extended their distribution from the Sinai Peninsula (origin of penetration) north-eastward into Palestine, Jordan, Lebanon, and Syria (Fig. 9B). Altogether, the majority (90 taxa) of this group extended their distribution into Palestine other than Egypt (Supplementary Table 2), while 35 taxa exhibited their distribution in Palestine, Jordan, Lebanon, and Syria besides Egypt. Perennial herbs (46 taxa) dominated other growth forms (32 annuals, 15 shrubs). *Allium tel-avivense, Origanum isthmicum, Haloxylon negevensis, Bellevalia zoharyi, Pimpinella cretica* var. *petraea*, *Onopordum alexandrinum,* and *Lupinus palaestinus* were examples of this group.
IV. East Mediterranean and Arabian Peninsula extensions: Thirty-eight taxa (26 perennial herbs, six shrubs, six annals) characterized this group, extending their distribution from Egypt eastward into Saudi Arabia and Yemen and north-eastward into East Mediterranean countries. A group of 28 taxa shared the distribution with Saudi Arabia, Palestine, and Jordan, including *Ferula sinaica, Atractylis mernephthae, Campanula dulcis, Silene hussoinii, Hypericum sinaicum, Stachys aegyptiaca, Astragalus intercedens, and Polygala sinaica* var. *glabrescens*.
V. Sudanian and Arabian Peninsula: This group consisted of 5 taxa (4 shrubs), extending their distribution from south and east Egypt in two directions: southward into Sudan and Eritrea (e, g., *Pancratium tortuosum* and *Taverniera aegyptiaca*) and eastward into Saudi Arabia and Yemen (e.g., *Echinops hussonii* and *Tribulus spurius*).
VI. North African, East Mediterranean, and Arabian extensions: *Echium longifolium,* *Erucaria microcarpa*, and *Echinops galalensis* constituted this group and were distributed outside the Egyptian borders in Libya, Palestine, Jordan, and Saudi Arabia.
VII. North African and Sudanian extensions: This group included the annual grass *Stipagrostis shawii*, which extended its distribution westward into Libya and southward into Sudan.
VIII. East Mediterranean, Sudanian, and Arabian Peninsula extensions: This group included the widely distributed perennial herb *Caudanthera sinaica*, which extended its distribution from East Egypt into Palestine, Saudi Arabia, and Sudan.

## Discussion

### Taxonomic differentiation and taxa verification

Over the past five decades, several publications (Täckholm 1974; El Hadidi and Fayed 1994/95; El Hadidi and Hosni 2000; Boulos 2009; Hosni et al. 2013; Abdelaal et al. 2018; El-Khalafy et al. 2021; Abdeaal, et al. 2020; Bedair et al. 2023) reported the numbers of endemic taxa in the flora of Egypt. However, an estimated definitive number is still inaccurate. The variations in estimating the total numbers of endemic taxa in publications over 50 years (1974–2023) were indicated in Supplementary Table (6). This study recognized that 70 taxa (3.3% of the total flora) belong to 28 families and 56 genera, of which 19 taxa are newly added. The number of endemic species fluctuated constantly as their known ranges grew or if they were taxonomically grouped with more widely distributed species. The possible major reasons for such differences can be attributed to (1) updated taxonomic investigations, (2) level of taxonomic ranks, (3) updated geographical ranges of taxa, (4) climate change, (5) human disturbances, and (6) habitat loss. Therefore, it is crucial to consider all the abovementioned reasons when comparing the results of different studies. Due to extremely hot desert environmental conditions (Hegazy and Lovett-Doust 2016; Abdelaal et al. 2020), similar trends of low endemism can be found in neighboring Libya (3.8%; Mahklouf and Etayeb (2019) Palestine (5.8%; Ighbareyeh et al. (2021), Saudi Arabia (10.7%; Attia et al. (2017) and Sudan (15%; Adam et al. 2020). In contrast, countries with wetter climates in the Mediterranean Basin and its islands, such as Morocco, Greece, and Italy, have higher levels of endemism such as Aedo et al. (2013) in Spain, Rankou et al. (2013) in Morocco, Dimopoulos et al. (2013) in Greece, and Fois et al. (2017) in Italy.

In the present study, the analysis of near-endemic recognized 181 taxa in 40 families and 111 genera, of which 76 are new additions. This number is much higher than the figures published by previous authors: 93 by Boulos (2009), 105 by Hosni et al. (2013), and 73 by Shaltout et al. (2018). More attention should be paid to the increasing numbers of near-endemic taxa, as they are often more vulnerable to extinction than widespread species. For conservation serious steps can be taken toward their conservation by protecting their habitats and reducing the threats they face. In addition to the reasons mentioned above, a few other factors could contribute to the increasing numbers of near-endemic taxa. For example, new technologies, such as DNA analysis, make identifying and distinguishing between closely related species easier. This could lead to the discovery of more near-endemic taxa that were previously overlooked. Additionally, as climate change continues to accelerate, more and more plant species will likely be forced to shift their ranges, which could lead to new near-endemic taxa. Recently, the status of several near-endemic taxa was altered due to intensive studies on endemism in adjacent countries (El-Khalafy et al. 2021). This investigation shared 38 taxa with other relevant studies (a complete list can be requested from authors).

Taxonomic differentiation affects the conservation of endemism in Egypt by providing more information about distribution ranges, conservation status, and habitat preferences and prioritizing conservation efforts (Joshi and Janarthanam 2004; Bonn et al. 2002) by identifying and protecting these unique and ecologically valuable plant species that are particularly vulnerable or threatened. The floristic analysis revealed that Asteraceae, Fabaceae, Caryophyllaceae, and Lamiaceae were the important families with the highest number of taxa and constituted the major components of both endemic and near-endemics. These families are among the most prevalent in North Africa's Mediterranean flora (White 1993). In terms of floristic diversity, these findings were in line with those of Walas and Taib (2022) in Morocco, Moghanloo et al. (2023) in Iran, and Georghiou and Delipetrou (2010) in Greece.

Compared to their abundance in Egypt’s flora, Poaceae (240 taxa; 11% of the total flora) was not among families harbor high numbers of endemic and/or near-endemic taxa. The low number of endemic grasses in Egypt coincides with those in neighboring arid countries such as Saudi Arabia (11 endemic types of grass out of 269 taxa; Chaudhary and Cope 1983), Libya (2 out of 229; Al-Sghair et al. 2019) and Palestine (5 out of 196; Ali-Shtayeh et al. 2022). The lower number of endemic and near-endemic taxa in Poaceae than in some other plant families can be attributed to their excellent dispersal ability, cosmopolitan distribution, and often wind pollination (Ricklefs 2008).

### Biological spectrum

Wang et al. (2003) indicated that plants have evolved different life forms to adapt to the different conditions along the hydrothermic gradient. Accordingly, trees are well-suited to warm and wet climates, while shrubs are better adapted to dry climates. These functional types have been used to describe plant adaptability to specific environmental conditions (Salama et al. 2015). No endemic trees were represented among growth forms, while shrubs and perennial herbs were best represented (44 taxa, 62.8%). Similarly, the composition of the biological spectrum of the near-endemics follows the same trend as in endemics where perennials dominate (124 taxa, 68.5%). In general, the paucity of trees and the high presence of shrubs in the flora of Egypt reflect its harsh climate, poor soils, and long history of human disturbance (Bedair et al. 2021). The annuals had the lowest proportion of endemic (26 out of 70 taxa, 37%) and near-endemic taxa (57 out of 181 taxa, 31.5%). The results of this study contradict those of El-Khalafy et al. (2021) and Abdelaal et al. (2018) who reported the dominance of annuals among other growth forms. In this study, the Mareotis sector of the Mediterranean coastal land (Mm) harbour the highest number of annual taxa, which can be attributed to high amounts of rainfall (annual mean 220–150 mm year^−1^; Bedair et al. 2023) and mild temperature (mean annual from 25.3 to 13.3 °C; Zahran and Willis 2009). In this study, the Mareotis sector of the Annual plants is characterized by their short life cycle, which means that they must produce seeds and complete their life cycle within a single growing season. In addition, the desert landscape of Egypt is frequently subjected to physical disturbances such as sandstorms, droughts, and uncontrolled anthropogenic activities, which are harmful environmental conditions not only for the growth of annuals but also for plant diversity and vegetation structure (Hussein et al. 2021).

*Medemia argun*, a known near-endemic tree from Egypt deserves special attention. The plant was known in Egypt since ancient times and was found in ancient Egyptian tombs, and it has not been recorded from Sudan since 1907 (Täckholm and Drar 1950). Boulos (1966) discovered some new individuals in Kurkur Oasis (Western Desert, Egypt). The leaves were used by the local people in making mats and as camel shacles. Whereas *Medemia argun* was considered extinct in Sudan by Uhl and Dransfield (1987), new populations were discovered by Gibbons and Spanner (1996). Recent populations for several *Medemia* palms were observed by Dina Ali and Rafik Khalil in Dungul Oasis (Boulos 2005), Western Desert, Egypt.

### Distribution patterns of taxa and habitat heterogeneity

The endemic plant species are often adapted to specific environmental conditions, such as soil type, topography, and climate, which restrict their distribution to a narrow geographic area. Grytnes and Vetaas (2002) indicated that topography can act as a barrier or facilitator for plant dispersal, forming distinct geographic patterns of endemism. Mountains, valleys, and other landforms can restrict the movement of plants, resulting in the isolation of populations and the development of unique species. Mountainous regions can act as water catchment areas, forming rivers, streams, and oases. These water sources support unique microhabitats and provide refuge for endemic plant species adapted to specific moisture requirements (Joshi and Janarthanam 2004).

In this study, the distribution patterns of endemic taxa indicated that 20 taxa (28.6% of the total endemics) showed a wide range of distribution that occurred in more than one OGU (multiple region endemic taxa, MRET), and 50 taxa (71.4% of the total) found in one OGU (single region endemic taxa, SRET). In both sets, most of these taxa were recorded from the mountainous Sinai (S) and the Mareotis sector of the Mediterranean coastal land (Mm). In the meantime, the sandy plains, and wadis (SPW, 24 taxa) and the rocky plains and mountains (RPM, 19 taxa) were the main habitats, forming more than 60% of the total endemic taxa. Similarly, higher numbers of near-endemic taxa were recorded from the Sinai and the Mediterranean OGUs. These findings were those obtained by Hosni et al. (2013), Shaltout et al. (2018), and El-Khalafy et al. (2021). Taxa with more extensive distribution ranges will be less vulnerable to extinction from natural or anthropogenic threats (Wulf et al. 2013) and have higher genetic diversity, making them more adaptable to changing conditions (Lavergne et al. 2004).

Local differentiation and reproductive isolation may be supported by impediments to gene flow among divergent populations caused by topographic complexity. Because of its large rocky massif, which supports several microhabitats, the South Sinai mountainous region has a wide diversity of plants (Dawood et al. 2022). This region’s high elevations prevent propagules from dispersing, which increases the number of endemic and near-endemic species (Ramadan et al. 2009; El-Keblawy 2014). *Phlomis aurea* Decne (Lamiaceae), an endangered endemic shrub growing in the mountainous areas of the southern Sinai Peninsula (El Hadidi and Hosni 2000), recently subjected to a dramatic decline in its population unless conservation measures should be taken for protection against anthropogenic and environmental stresses (Serag et al. 2018).

The Mediterranean Basin is the world’s second-largest biodiversity hotspot (Lopez-Alvarado and Farris 2022). The vegetation is limited to microenvironments such as wadis, runnels, and depressions along Egypt’s Mediterranean coast, where runoff water accumulates and provides enough moisture for plant growth (Salama et al. 2013). In a recent study on the Mediterranean endemic taxa in the Egyptian flora, Bedair et al. (2023) recognized 15 habitats supported the occurrence of 65 Mediterranean endemic taxa in Egypt, where the most characterized habitats were the non-saline downturns followed by the coastal dunes. Compared to other regions of Egypt, the Mediterranean region is characterized by a very high degree of endemism since many species are limited to a single or small number of sites in sandy areas, isolated mountain ranges, islands with peculiar soils, or rocky grounds (Zahran 2010). Over the past three decades, the western Mediterranean coast of Egypt was subjected to uncontrolled human disturbances and sprawling urbanization projects (i.e., the establishment of tourist summer resorts), which significantly negatively affected the natural habitats supporting characteristic vegetation and flora (Bedair et al. 2023; Halmy 2019). Consequently, these impacts can lead to declines in population size and range and a decrease in genetic diversity and gene flow for both endemic and near-endemic taxa (Halmy et al. 2015a, b). In this context, most of the calcareous sand dunes extended along the western Mediterranean coast become threatened habitats with endangered flora (Ahmed et al. 2014). *Ebenus armitagei* Schweinf. & Taub., *Nigella arvensis* L. subsp. taubertii (Brand) Maire, *Plantago crypsoides* Boiss., and *Centaurea glomerata* Vahl. were considered threatened near-endemic taxa that characterized this ecosystem.

### Floristic regionalization

#### Endemic taxa

Five main floristic groups (I–V) have resulted after the application of cluster analysis on the occurrences of 70 endemic taxa in the 14 studied OGUs, each characterized by certain OGUs: (I) included the Sinai Peninsula (Di, S) and the Suez Gulf sector of the Red Sea coastal plains (Rz), (II) included OGUs of the Mediterranean coast (Mm, Ms), (III) included the Eastern Desert (Da, Dg) and the Nile Valley (Nv), (IV) included the Western Desert (Dl, Ol, On) and the Nubian Nile (Nn), and (V) included Gebel Elba highlands (Ge) and the Red Sea coast (Ra). Using hierarchical clustering and indicator values studies, Abdelaal et al. (2020) determined six biogeographical sectors and six subsectors based on the presence-absence matrix of 140 endemic taxa. The sectors were Libo-Nubian, Nilotic, Marioutico-Arishian, Sinaico-Arabian, Elbanian, and Suezian, while the subsectors were Deltaic, Fayoumian, Marioutic, Arishian, Sinaic, and Arabian. Abdelaal et al. (2018) divided Egypt into three main groups: I (Eastern Egypt and the Sinai Peninsula), II (Western Egypt including the Western Desert and Oases), and III (Middle Egypt including the Nile lands). The results of this investigation were partially in agreement with both studies. The latter divisions ignored the Mediterranean and Gebel Elba regions as distinct phytogeographic territories with remarkable flora (Abd El-Ghani et al. 2017a).

Although the Sinai Peninsula and the Eastern Desert of Egypt are two distinct geographical regions, they are also closely related. Geologically, the Sinai Peninsula and the Eastern Desert are part of the Arabian-Nubian Shield (Said 1962), a geological formation characterized by ancient Precambrian rocks. The two regions also share a similar climate characterized by hot, dry summers and mild winters. However, the Sinai Peninsula receives more precipitation than the Eastern Desert due to its higher elevation and proximity to the Mediterranean Sea (Abd El-Ghani et al. 2017b; Salama et al. 2013). On the other hand, the relationship between Gebel Elba and the Eastern Desert of Egypt is complex and multifaceted. Gebel Elba is physically and ecologically distinct from the surrounding desert but is also an integral part of the region. It provides water, habitat, and recreation for the people and wildlife of the Eastern Desert of Egypt (Shaltout et al. 2016). Based on the information above, the recognized groups of endemic taxa presented in this study were more realistic and incongruent with the eco-geographical and floristic integration between the regions included. This study proved the insignificant relationship between the Gebel Elba group (group V) and the Eastern Desert (group III), which means high dissimilarity between the compositions of endemic flora. In the meantime, the Mediterranean group (group II) and the Eastern Desert group (group III) were insignificantly correlated. Such relations contradict the classifications of Abdalaal et al. (2018 and 2020).

#### Near-endemic taxa

When comparing the distribution patterns of the near-endemic taxa outside Egypt, Hosni et al. (2013) grouped their 105 taxa into four main territories: (1) North-eastern territory included Sinai, Galala desert, S and C Palestine with slight extensions to SW Jordan and NW Arabia, (2) Northwestern territory included NW Egypt to NE Libya, (3) South-western territory included SW Egypt, SE Libya, and NW Sudan, and (4) South-eastern territory included SE Egypt and Sudan, slightly extending to SW Arabia. According to Hosni et al. (2013), the majority (92%; 56 taxa out of the 61 near-endemic taxa were mainly distributed in the north-eastern and the northwestern territories. Due to differences in numbers and locations, comparing such four territories with the eight main groups indicated in this investigation took much work. This can be reflected by the low Jaccard’s similarity index (0.44) between this investigation and the latter. However, specific taxa showed similar distribution, especially in the northwestern and South-western territories, such as *Crepis libyca, Silene biappendiculata,* and *Carthamus glaucus *subsp. *alexandrinus *in the former territory, and *Stipagrostis shawii* in the latter. Due to recent taxonomic revisions, 47 taxa were excluded from the list of near-endemics (recorded in previous studies), which either became synonyms to other taxa, their distribution became wider, or their status has been changed (full lists can be requested from authors). Such taxa that became endemic included, amongst others, *Allium crameri*, *Convolvulus schimperi*, *Ducrosia ismaelis*, *Euphorbia punctata*, and *Nepeta septemcrenata*. In addition, *the present study did not confirm Nigella arvensis* var. *beersherensis *and *Euphorbia bivonae* var. *sinaica*.

## Conclusions

The present study presented a comprehensive compiled list of plant endemism in the Egyptian flora. The current status consisted of 70 endemic and 181 near-endemic taxa, the majority growing in sandy plains and wadis (SPW) and the rocky plains and mountains (RPM). Considering the critical revision of their geographical distribution ranges in 14 Operational Geographical Units (OGUs) inside (for endemics) and outside (for near-endemics), 19 endemics and 76 near-endemics are newly added taxa. Several amendments to those previously known endemic and near-endemic taxa were indicated. The mountainous Sinai (S) and the Mareotis sector of the Mediterranean coastal land (Mm) proved they harbor the largest number of endemic and near-endemic taxa. Although Poaceae are best represented in the Egyptian flora, other important families included the higher numbers of endemics and near-endemics. The critically endangered endemic taxa were the largest among other IUCN conservation categories used, mainly from Caryophyllaceae and Lamiaceae.

In contrast, Asteraceae and Fabaceae had the highest numbers in the extinct (EX) taxa. Hierarchical cluster analysis of the endemic taxa dataset revealed five main floristic groups that correspond to ‘floristic regionalization’; each included one or more OGUs of this study. In the meantime, eight possible routes of extensions of near-endemic taxa were provided. Two major pathways of extensions outside Egypt were proposed: East Mediterranean extension (93 taxa) and East Mediterranean and Arabian Peninsula (38 taxa).

### Supplementary Information

Supplementary Material 1.

Supplementary Material 2.

Supplementary Material 3.

Supplementary Material 4.

Supplementary Material 5.

Supplementary Material 6.

Supplementary Material 7.

Supplementary Material 8.

## Data Availability

All data generated and analysed during this study are included in this article and its additional information files.

## Abbreviations

OGUs: Operational Geographical Units
T: Trees
S: Perennial herbs
A: Annuals
Ds: Species differentiation index
Dg: Genus differentiation index
Dsf: Species–family differentiation index
SRET: Single-region endemic taxa
MRET: Multiple-region endemic taxa
SPW: Sandy plains and wadis
RPM: Rocky plains and mountains
AL: Arable lands
MGCB: Moist ground and canal banks
SG: Stony ground
CSP: Coastal sandy plains, and dry DSM salt marshes
CR: Critically endangered
DD: Data deficient
EN: Endangered
EX: Extinct
LC: Least concern
NT: Near threatened
VU: Vulnerable
PCA: Principal components analysis
GF: Growth forms
