# Supplementary material for: New perspectives, additions, and amendments to plant endemism in a North African flora
Source: Bot Stud. 2024 Jul 16;65:21. doi: 10.1186/s40529-024-00428-w (PMC11252113; doi:10.1186/s40529-024-00428-w)
Supplement: Supplementary file 1 — Supplementary Material 1. [file 40529_2024_428_MOESM1_ESM.doc]

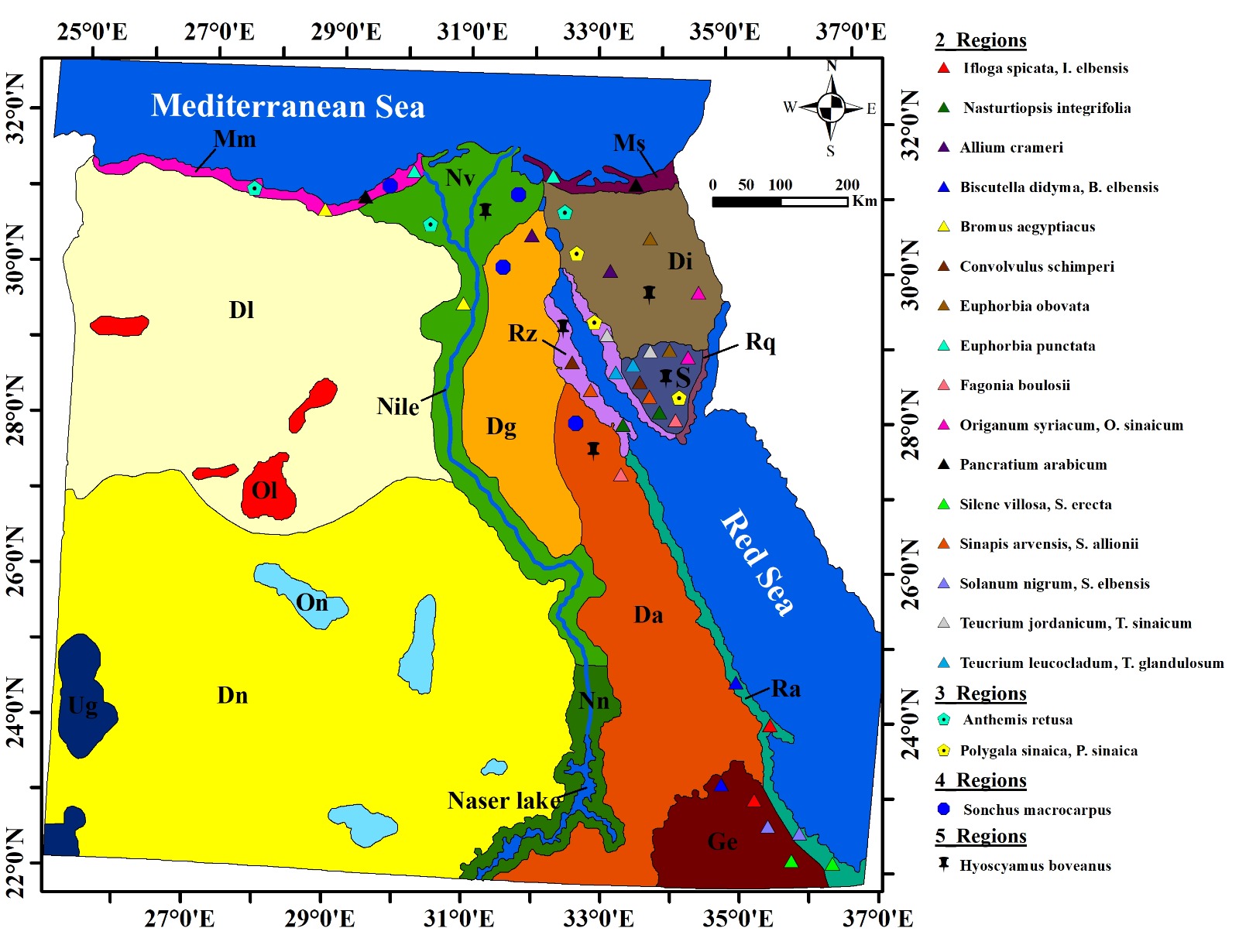


**A**


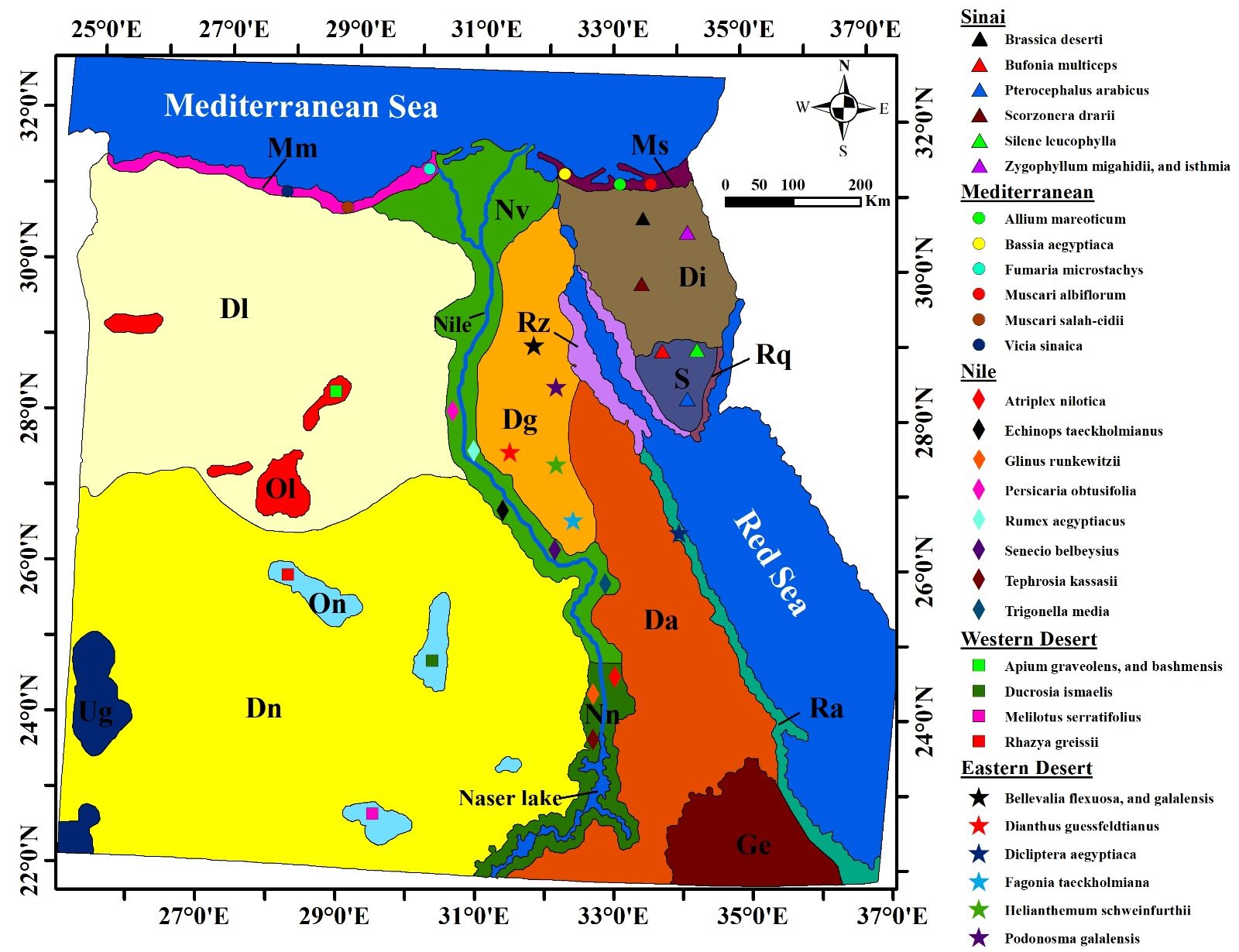


**B**


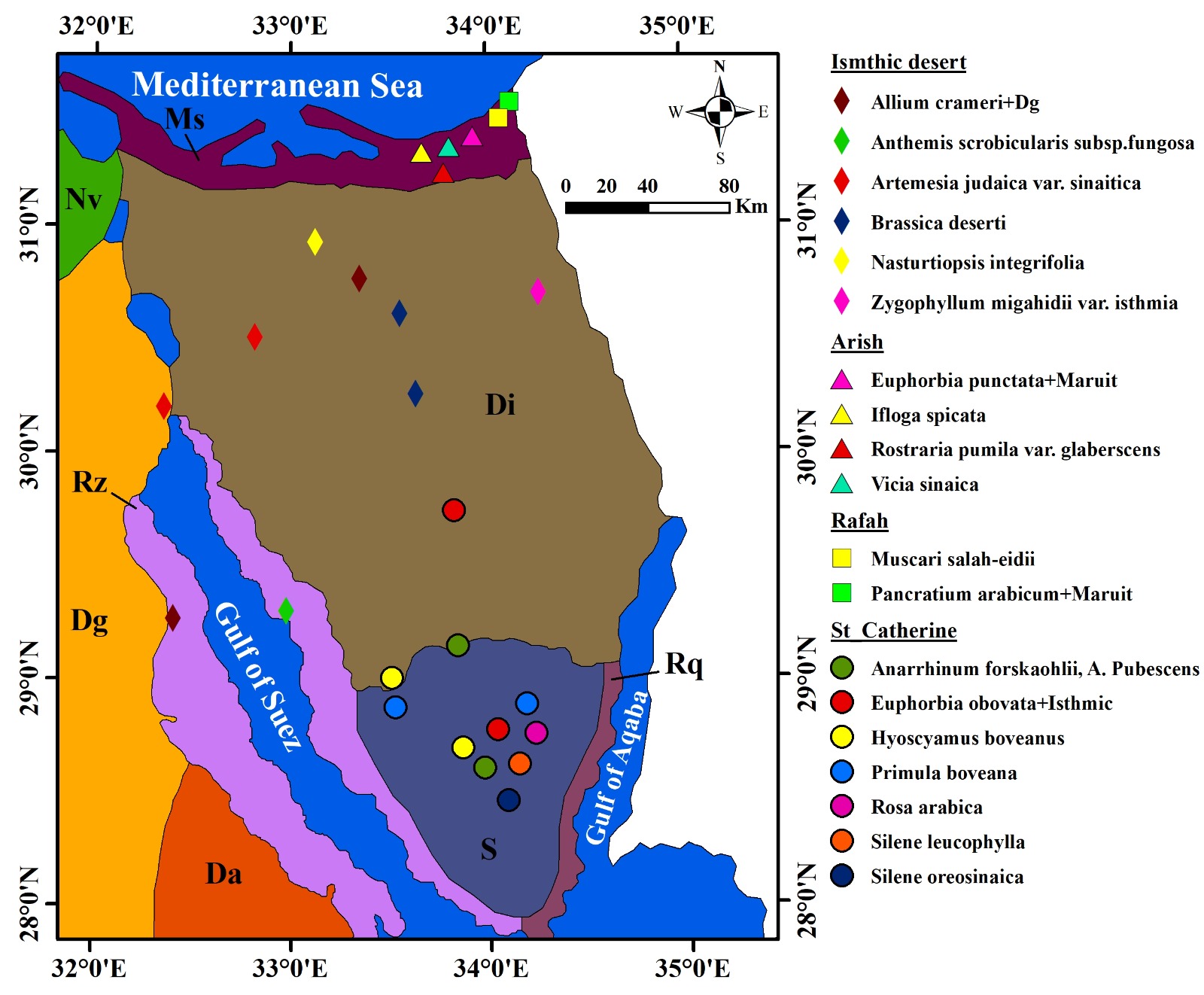


**C**

**Supplementary Fig. 1** Distribution maps of some endemic taxa, **(A)** in the OGUs (Regions) they occur, **(B)** in one OGU, and **(C)** in Sinai OGU. For abbreviations of OGUs, see Fig. 1.
