# Supplementary material for: New perspectives, additions, and amendments to plant endemism in a North African flora
Source: Bot Stud. 2024 Jul 16;65:21. doi: 10.1186/s40529-024-00428-w (PMC11252113; doi:10.1186/s40529-024-00428-w)
Supplement: Supplementary file 2 — Supplementary Material 2. [file 40529_2024_428_MOESM2_ESM.doc]

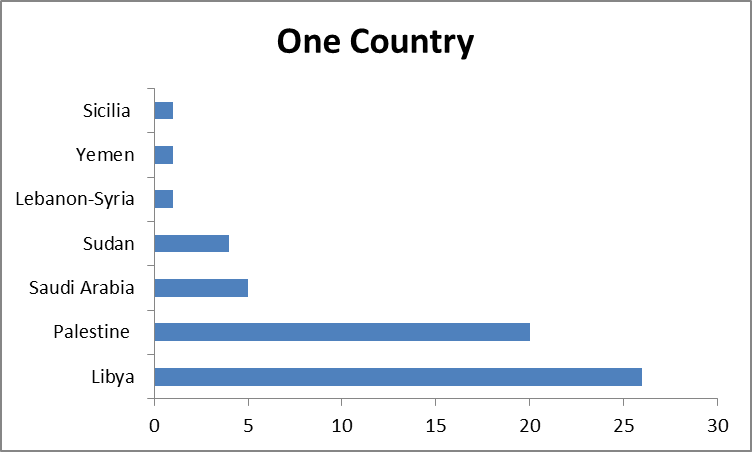


**A**

**
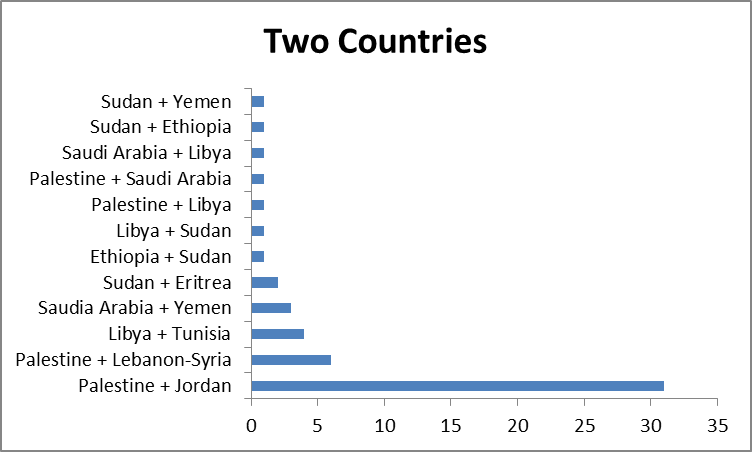
**

**B**


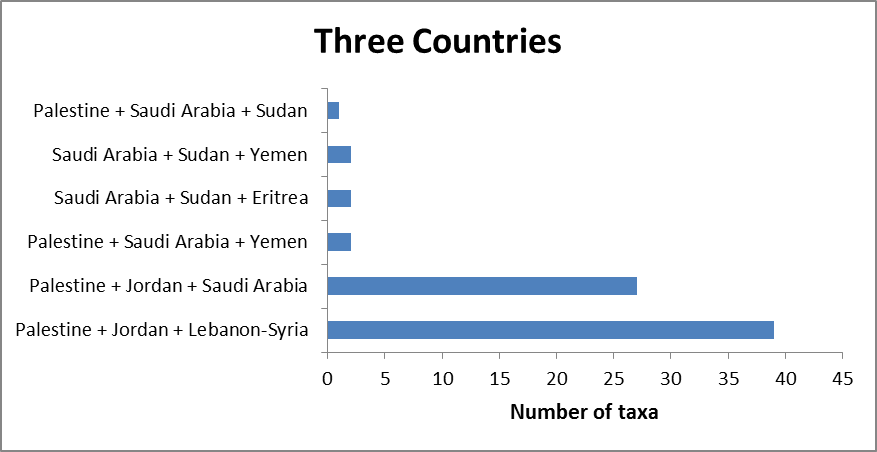


**C**

**Supplementary Fig. 2** **A, B and C** Distribution of shared numbers of near-endemic taxa in neighbouring countries (outside Egypt)
