# Supplementary material for: New perspectives, additions, and amendments to plant endemism in a North African flora
Source: Bot Stud. 2024 Jul 16;65:21. doi: 10.1186/s40529-024-00428-w (PMC11252113; doi:10.1186/s40529-024-00428-w)
Supplement: Supplementary file 3 — Supplementary Material 3. [file 40529_2024_428_MOESM3_ESM.doc]

**Supplementary Table 1. Distribution of 70 endemic taxa in the 14 OGUs of the present study. Groups (I-V) are those referred to in Figs 6 & 7. For full names of OGUs, see Figure (1); for other abbreviations, see text. Taxa in bold and preceded by an asterisk (*) are the Indicator taxa with significant p-values obtained from Indicator Species analysis. **= p-values significant at 0.01, *= **= p-values significant at 0.05.**

| Floristic groups | Group (I) | | | Group (II) | | Group (III) | | | Group (VI) | | | | Group (V) | | Habitat | IUCN category | GF |
| --- | --- | --- | --- | --- | --- | --- | --- | --- | --- | --- | --- | --- | --- | --- | --- | --- | --- |
| Number of taxa | 28 | | | 20 | | 17 | | | 8 | | | | 5 | |
| Operational Geographical Units (OGUs) | S | Di | Rz | Ms | Mm | Nv | Dg | Da | Nn | Dl | Ol | On | Ge | Ra |
| Multiple region endemic taxa (MRET) | | | | | | | | | | | | | | | | | |
| *Allium crameri* Asch. & Boiss. | - | + | - | - | - | - | + | - | - | - | - | - | - | - | RPM | EN | PH |
| *Anthemis retusa* Delile | - | - | - | - | + | + | - | - | - | + | - | - | - | - | AL | NE | A |
| **Biscutella* *didyma* L. var. *elbensis* (Chrtek) El Naggar (V) | - | - | - | - | - | - | - | - | - | - | - | - | + | + | RPM | CR | A |
| *Bromus aegyptiacus* Tausch. | - | - | - | - | + | + | - | - | - | - | - | - | - | - | AL | VU | A |
| *Convolvulus schimperi* Boiss. | + | - | + | - | - | - | - | - | - | - | - | - | - | - | SPW | EN | PH |
| *Euphorbia obovata* Decne. | + | + | - | - | - | - | - | - | - | - | - | - | - | - | SPW | VU | S |
| **Euphorbia punctata* Delile (II) | - | - | - | + | + | - | - | - | - | - | - | - | - | - | AL | CR | A |
| *Fagonia boulosii* Hadidi | + | - | - | - | - | - | - | + | - | - | - | - | - | - | SPW | VU | S |
| **Hyoscyamus boveanus* (Dunal) Asch. & Schweinf. (I) | + | + | + | - | - | + | - | + | - | - | - | - | - | - | SPW | LC | PH |
| **Ifloga spicata* (Forssk.) Sch.Bip. subsp. *elbaensis* Chrtek (V) | - | - | - | - | - | - | - | - | - | - | - | - | + | + | SPW | CR | A |
| *Nasturtiopsis integrifolia* (Boulos) Abdel Khalik & F.T.Bakker | + | - | + | - | - | - | - | - | - | - | - | - | - | - | SPW | CR | A |
| *Origanum syriacum* L subsp. *sinaicum* (Boiss.) Greuter & Burdet | + | + | - | - | - | - | - | - | - | - | - | - | - | - | SG | EN | S |
| **Pancratium arabicum* Sickenb. (II) | - | - | - | + | + | - | - | - | - | - | - | - | - | - | SPW | EN | PH |
| ***Polygala sinaica* Botsch. var. *Sinaica*  (I) | + | + | + | - | - | - | - | - | - | - | - | - | - | - | RPM | VU | S |
| **Silene villosa* Forssk. var. *erecta* Täckh. & Boulos (V) | - | - | - | - | - | - | - | - | - | - | - | - | + | + | SPW | EN | A |
| *Sinapis arvensis* subsp. *allionii* (Jacq.) Baillarg. | - | - | - | - | + | + | - | - | - | - | - | - | - | - | AL | VU | A |
| **Solanum nigrum* L. var. *elbaensis* Täckh. & Boulos (V) | - | - | - | - | - | - | - | - | - | - | - | - | + | + | SPW | CR | A |
| **Sonchus macrocarpus* Boulos & C.Jeffrey (III) | - | - | - | - | + | + | + | + | - | - | - | - | - | - | MDCB | VU | A |
| *Teucrium jordanicum* (Danin) Faried var. *sinaicum* (Danin) Faried | + | - | + | - | - | - | - | - | - | - | - | - | - | - | RPM | VU | S |
| *Teucrium leucocladum* Boiss. var. *glandulosum* Danin | + | - | + | - | - | - | - | - | - | - | - | - | - | - | RPM | VU | S |
| Single region endemic taxa (SRET) | | | | | | | | | | | | | | | | | |
| *Allium mareoticum* Bornm. & Gauba | - | - | - | - | + | - | - | - | - | - | - | - | - | - | RPM | CR | PH |
| *Anarrhinum forskaohlii* (J.F.Gmel.) Cufod. subsp. *pubescens* D.A.Sutton | + | - | - | - | - | - | - | - | - | - | - | - | - | - | RPM | CR | PH |
| *Anthemis microsperma* Boiss. & Kotschy | - | - | - | - | + | - | - | - | - | - | - | - | - | - | SPW | VU | A |
| *Apium graveolens* var. *bashmensis* H.Hosni | - | - | - | - | - | - | - | - | - | - | + | - | - | - | MDCB | EN | A |
| *Astragalus fresenii* Decne. | + | - | - | - | - | - | - | - | - | - | - | - | - | - | SPW | EN | PH |
| *Atractylis carduus* var. *marmarica* Täckh. & Boulos | - | - | - | - | + | - | - | - | - | - | - | - | - | - | CSP | EN | PH |
| *Atriplex nilotica* Sukhor. | - | - | - | - | - | - | - | - | + | - | - | - | - | - | MDCB | DD | S |
| *Ballota kaiseri* Täckh. | + | - | - | - | - | - | - | - | - | - | - | - | - | - | SG | CR | PH |
| *Bassia aegyptiaca* Turki, El Shayeb & F.Shehata | - | - | - | - | + | - | - | - | - | - | - | - | - | - | SPW | DD | A |
| *Bellevalia flexuosa* var. *galalensis* Täckh. & Drar ex Täckh. & Boulos | - | - | - | - | - | - | + | - | - | - | - | - | - | - | RPM | CR | PH |
| *Brassica deserti* Danin & Hedge | - | + | - | - | - | - | - | - | - | - | - | - | - | - | SG | VU | A |
| *Bufonia multiceps* Decne. | + | - | - | - | - | - | - | - | - | - | - | - | - | - | SG | EN | PH |
| *Dianthus guessfeldtianus* Muschl. | - | - | - | - | - | - | + | - | - | - | - | - | - | - | SG | CR | PH |
| *Dicliptera aegyptiaca* E. Shamso | - | - | - | - | - | - | - | - | - | - | - | - | - | + | RPM | DD | PH |
| *Ducrosia ismaelis* Asch. | - | - | - | - | - | - | - | - | - | - | - | + | - | - | SPW | EX | PH |
| *Echinops taeckholmianus* Amin | - | - | - | - | - | + | - | - | - | - | - | - | - | - | SPW | DD | PH |
| *Euphorbia sanctae-catharinae* Fayed | + | - | - | - | - | - | - | - | - | - | - | - | - | - | SPW | CR | PH |
| *Fagonia taeckholmiana* Hadidi | - | - | - | - | - | - | + | - | - | - | - | - | - | - | SPW | EX | PH |
| *Fumaria microstachys* Kralik ex Hausskn. | - | - | - | + | - | - | - | - | - | - | - | - | - | - | AL | CR | A |
| *Glinus runkewitzii* Täckh. & Boulos | - | - | - | - | - | - | - | - | + | - | - | - | - | - | MDCB | EX | A |
| *Helianthemum schweinfurthii* Grosser | - | - | - | - | - | - | + | - | - | - | - | - | - | - | SPW | CR | S |
| *Ifloga spicata* (Forssk.) Sch.Bip. subsp. *hadidii* (Fayed & Zareh) Greuter | + | - | - | - | - | - | - | - | - | - | - | - | - | - | SPW | DD | A |
| *Limonium mareoticum* El Garf in El Hadidi & Fayed | - | - | - | - | + | - | - | - | - | - | - | * | - | - | DSM | CR | PH |
| *Limonium sinuatum*  (L.) Mill. subsp. *romanum* Täckh. & Boulos | - | - | - | - | + | - | - | - | - | - | - | - | - | - | MDCB | EX | PH |
| *Melilotus serratifolius* Täckh. & Boulos | - | - | - | - | - | - | - | - | - | - | - | + | - | - | AL | DD | A |
| *Micromeria serbaliana* Danin & Hedge | + | - | - | - | - | - | - | - | - | - | - | - | - | - | RPM | CR | PH |
| *Muscari albiflorum* (Täckh. & Boulos) Hosni | - | - | - | - | + | - | - | - | - | - | - | - | - | - | RPM | CR | PH |
| *Muscari salah-eidii* (Täckh. & Boulos) Hosni | - | - | - | + | - | - | - | - | - | - | - | - | - | - | RPM | EX | PH |
| *Nepeta septemcrenata* Ehrenb. ex Benth. | + | - | - | - | - | - | - | - | - | - | - | - | - | - | SG | CR | PH |
| *Persicaria obtusifolia* (Täckh. & Boulos) Greuter & Burdet | - | - | - | - | - | + | - | - | - | - | - | - | - | - | MDCB | EX | PH |
| *Phlomis aurea* Decne. | + | - | - | - | - | - | - | - | - | - | - | - | - | - | SG | EN | PH |
| *Podonosma galalensis* Schweinf. ex Boiss. | - | - | - | - | - | - | + | - | - | - | - | - | - | - | RPM | CR | PH |
| *Primula boveana* Decne. Ex Duby | + | - | - | - | - | - | - | - | - | - | - | - | - | - | RPM | CR | PH |
| *Pterocephalus arabicus* Boiss. | + | - | - | - | - | - | - | - | - | - | - | - | - | - | RPM | CR | S |
| *Rhazya greissii* Täckh.& Boulos | - | - | - | - | - | - | - | - | - | - | - | + | - | - | SPW | EX | S |
| *Rosa arabica* Crép. | + | - | - | - | - | - | - | - | - | - | - | - | - | - | RPM | CR | S |
| *Rumex aegyptiacus* L. | - | - | - | - | - | + | - | - | - | - | - | - | - | - | MDCB | EN | A |
| *Scorzonera drarii* Tackh. | - | + | - | - | - | - | - | - | - | - | - | - | - | - | AL | EX | PH |
| *Senecio belbeysius* Delile | - | - | - | - | - | + | - | - | - | - | - | - | - | - | AL | EX | A |
| *Silene apetala* Willd. var*. glabrata* A. Hossny & E. Shamso | - | - | - | - | + | - | - | - | - | - | - | - | - | - | SPW | CR | A |
| *Silene biappendiculata* Ehrenb. ex Rohrb. var. *granulata* E.Shamso | - | - | - | - | + | - | - | - | - | - | - | - | - | - | SPW | LC | A |
| *Silene leucophylla* Boiss. | + | - | - | - | - | - | - | - | - | - | - | - | - | - | RPM | CR | PH |
| *Silene oreosinaica* Chowdhuri | + | - | - | - | - | - | - | - | - | - | - | - | - | - | RPM | CR | PH |
| *Silene* *odontopetala* Fenzl var. *congesta* Boiss. | + | - | - | - | - | - | - | - | - | - | - | - | - | - | RPM | CR | PH |
| *Tephrosia kassasii* Boulos | - | - | - | - | - | - | - | - | + | - | - | - | - | - | SPW | EX | PH |
| *Thesium humile* Vahl var. *maritima* (N.D.Simpson) Sa’ad | - | - | - | - | + | - | - | - | - | - | - | - | - | - | CSP | VU | A |
| *Trigonella media* Delile ex Urb. | - | - | - | - | - | + | - | - | - | - | - | - | - | - | AL | DD | A |
| *Veronica anagalloides* Guss. subsp. *taeckholmiorum* Chrtek & Osb.-Kos. | - | - | - | - | + | - | - | - | - | - | - | - | - | - | MDCB | DD | A |
| *Vicia sinaica* Boulos | - | - | - | + | - | - | - | - | - | - | - | - | - | - | SPW | EX | A |
| *Zygophyllum migahidii* Hadidi var. *isthmia* A.Hosny | - | + | - | - | - | - | - | - | - | - | - | - | - | - | SPW | CR | PH |
