# Supplementary material for: New perspectives, additions, and amendments to plant endemism in a North African flora
Source: Bot Stud. 2024 Jul 16;65:21. doi: 10.1186/s40529-024-00428-w (PMC11252113; doi:10.1186/s40529-024-00428-w)
Supplement: Supplementary file 4 — Supplementary Material 4. [file 40529_2024_428_MOESM4_ESM.doc]

**Supplementary Table 2.** Distribution of near-endemic taxa in Egypt (including Sinai) and some adjacent countries in Africa and Asia, together with their families, growth forms and degrees of threat in Egypt. EG= Egypt, SU= Sudan, LB= Libya, T= Tunisia, ER=Eritrea, P= Palestine, L-S= Lebanon-Syria, SA= Saudi Arabia, J= Jordan, Y= Yemen. GF= Growth form: A= Annual, PH= Perennial Herb, S= Shrub, T= Tree, += present, *= new additions, -= absent. Families and taxa are alphabetically arranged.

| Continent |  |  | | Africa | | | | | | | | | | | | Asia | | | | | | | |
| --- | --- | --- | --- | --- | --- | --- | --- | --- | --- | --- | --- | --- | --- | --- | --- | --- | --- | --- | --- | --- | --- | --- | --- |
| Families | GF | Taxa | | EG | | Sinai | | SU | | LB | | T | | ER | | P | L-S | | SA | | J | | Y |
| (I) North African extension | | | | | | | | | | | | | | | | | | | | | | | |
| Egypt + one country | | | | | | | | | | | | | | | | | | | | | | | |
| Amaryllidaceae | PH | *Allium barthianum* Asch. & Schweinf. | + | |  | |  | | + | |  | |  | |  | |  |  | |  | |  | |
| Amaryllidaceae | PH | **Allium blomfieldianum* Asch. & Schweinf. | + | |  | |  | | + | |  | |  | |  | |  |  | |  | |  | |
| Apiaceae | A | *Bupleurum nanum* Poir. | + | |  | |  | | + | |  | |  | |  | |  |  | |  | |  | |
| Apiaceae | PH | *Ferula marmarica* Asch. & Taub. | + | |  | |  | | + | |  | |  | |  | |  |  | |  | |  | |
| Asparagaceae | PH | *Bellevalia sessiliflora* (Viv.) Kunth | + | |  | |  | | + | |  | |  | |  | |  |  | |  | |  | |
| Asteraceae | A | *Carthamus glaucus* subsp. *alexandrinus* (Boiss. & Heldr.) Hanelt | + | |  | |  | | + | |  | |  | |  | |  |  | |  | |  | |
| Asteraceae | A | *Carthamus mareoticus* Delile | + | |  | |  | | + | |  | |  | |  | |  |  | |  | |  | |
| Asteraceae | PH | *Crepis libyca* (Pamp.) Babc. | + | |  | |  | | + | |  | |  | |  | |  |  | |  | |  | |
| Asteraceae | A | **Anthemis eliezrae*Eig |  | | + | |  | |  | |  | |  | | + | |  |  | |  | |  | |
| Boraginaceae | A | *Nonea vivianii* DC. | + | |  | |  | | + | |  | |  | |  | |  |  | |  | |  | |
| Brassicaceae | A | *Enarthrocarpus pterocarpus* (Pers.) DC. | + | |  | |  | | + | |  | |  | |  | |  |  | |  | |  | |
| Brassicaceae | PH | *Pseuderucaria clavata* (Boiss. & Reut.) O. E. Schulz subsp. *clavata* | + | |  | |  | |  | |  | |  | | + | |  |  | |  | |  | |
| Caryophyllaceae | PH | *Herniaria cyrenaica* F.Herm. | + | |  | |  | | + | |  | |  | |  | |  |  | |  | |  | |
| Caryophyllaceae | A | *Silene biappendiculata* Ehrh. ex Rohrb. | + | |  | |  | | + | |  | |  | |  | |  |  | |  | |  | |
| Caryophyllaceae | S | **Silene fruticosa* L. subsp. *cyrenaica* Bég. et A. Vacc. | + | |  | |  | | + | |  | |  | |  | |  |  | |  | |  | |
| Caprifoliaceae | A | *Valerianella pterovichii* Asch. | + | |  | |  | | + | |  | |  | |  | |  |  | |  | |  | |
| Cistaceae | PH | *Helianthemum crassifolium* subsp. *sphaerocalyx* (Gauba & Janch.) Maire | + | |  | |  | | + | |  | |  | |  | |  |  | |  | |  | |
| Euphorbiaceae | A | *Euphorbia parvula* Delile | + | |  | |  | | + | |  | |  | |  | |  |  | |  | |  | |
| Fabaceae | S | *Ebenus armitagei* Schweinf. & Taub. | + | |  | |  | | + | |  | |  | |  | |  |  | |  | |  | |
| Lamiaceae | S | **Teucrium decaisnei* C.Presl | + | |  | |  | | + | |  | |  | |  | |  |  | |  | |  | |
| Papaveraceae | A | *Hypecoum aequilobum* Viv. | + | |  | |  | | + | |  | |  | |  | |  |  | |  | |  | |
| Plantaginaceae | PH | **Veronica catenata* subsp. *pseudocatenata* Chrtek & Osb.-Kos. | + | |  | |  | | + | |  | |  | |  | |  |  | |  | |  | |
| Plumbaginaceae | PH | **Limonium tubiflorum* (Delile) Kuntze | + | |  | |  | | + | |  | |  | |  | |  |  | |  | |  | |
| Plumbaginaceae | PH | **Limonium zanonii* (Pamp.) Domina | + | |  | |  | | + | |  | |  | |  | |  |  | |  | |  | |
| Resedaceae | A | **Reseda lutea* L. subsp*. petrovichiana (*Müll.Arg.) Jafri | + | |  | |  | | + | |  | |  | |  | |  |  | |  | |  | |
| Rubiaceae | A | *Valantia columella* (Ehrenb. ex Boiss.) Bald. | + | |  | |  | | + | |  | |  | |  | |  |  | |  | |  | |
| Scrophulariaceae | S | *Verbascum letourneuxii* Asch. | + | |  | |  | | + | |  | |  | |  | |  |  | |  | |  | |
| Brassicaceae | S | **Zilla spinosa* (L.) Prantl subsp. *biparmata* (O.E.Schulz) Maire & Weiller | + | |  | |  | | + | |  | |  | |  | |  |  | |  | |  | |
| Egypt + two countries | | | | | | | | | | | | | | | | | | | | | | | |
| Apiaceae | A | **Daucus syrticus* Murb. | + | |  | |  | | + | | + | |  | |  | |  |  | |  | |  | |
| Asteraceae | A | *Centaurea glomerata* Vahl | + | |  | |  | | + | | + | |  | |  | |  |  | |  | |  | |
| Fabaceae | S | **Lotus polyphyllos* E.D.Clarke | + | |  | |  | | + | | + | |  | |  | |  |  | |  | |  | |
| Ranunculaceae | A | **Nigella arvensis* L. subsp. *taubertii* (Brand) Maire | + | |  | |  | | + | | + | |  | |  | |  |  | |  | |  | |
| (II) Sudanian extension | | | | | | | | | | | | | | | | | | | | | | | |
| Egypt + one country | | | | | | | | | | | | | | | | | | | | | | | |
| Arecaceae | T | *Medemia argun* (Mart.) Württemb. ex H.Wendl. | + | |  | | + | |  | |  | |  | |  | |  |  | |  | |  | |
| Cucurbitaceae | PH | **Coccinia abyssinica* (Lam.) Cogn. + Ethiopia | + | |  | | + | |  | |  | |  | |  | |  |  | |  | |  | |
| Fabaceae | A | *Lotus nubicus* Hochst. ex Baker | + | |  | | + | |  | |  | |  | |  | |  |  | |  | |  | |
| Plantaginaceae | PH | *Veronica anagallis-aquatica* var. *nilotica* R.Uechr. | + | |  | | + | |  | |  | |  | |  | |  |  | |  | |  | |
| Plantaginaceae | PH | *Veronica scardica* subsp. *africana* Chrtek & Osb.-Kos. | + | |  | | + | |  | |  | |  | |  | |  |  | |  | |  | |
| Egypt + two countries | | | | | | | | | | | | | | | | | | | | | | | |
| Cyperaceae | PH | **Cyperus microbolbos* C.B.Clarke | + | |  | | + | |  | |  | | + | |  | |  |  | |  | |  | |
| Fabaceae | S | *Lotus hebranicus* Hochst. ex Brand | + | |  | | + | |  | |  | | + | |  | |  |  | |  | |  | |
| (III) East Mediterranean extension | | | | | | | | | | | | | | | | | | | | | | | |
| Egypt + one country | | | | | | | | | | | | | | | | | | | | | | | |
| Amaryllidaceae | PH | **Allium curtum* subsp. *palaestinum* Feinbrun |  | | + | |  | |  | |  | |  | | + | |  |  | |  | |  | |
| Amaryllidaceae | PH | **Allium papillare* Boiss. |  | | + | |  | |  | |  | |  | | + | |  |  | |  | |  | |
| Amaryllidaceae | PH | *Allium tel-avivense* Eig | + | |  | |  | |  | |  | |  | | + | |  |  | |  | |  | |
| Asparagaceae | PH | *Muscari eburneum* (Eig & Feinbrun) D.C.Stuart | + | |  | |  | |  | |  | |  | | + | |  |  | |  | |  | |
| Asparagaceae | PH | *Muscari longistylum* (Täckh.& Boulos) Hosni |  | | + | |  | |  | |  | |  | | + | |  |  | |  | |  | |
| Asteraceae | A | *Atractylis boulosii* Täckh. |  | | + | |  | |  | |  | |  | | + | |  |  | |  | |  | |
| Asteraceae | PH | **Atractylis carduus* (Forssk.) C.Chr. var. *glabrescens* (Boiss.) Täckh. & Boulos |  | | + | |  | |  | |  | |  | | + | |  |  | |  | |  | |
| Lamiaceae | S | *Origanum isthmicum* Danin |  | | + | |  | |  | |  | |  | | + | |  |  | |  | |  | |
| Lamiaceae | PH | **Pseudodictamnus damascenus* (Boiss.) Salmaki & Siadati | + | |  | |  | |  | |  | |  | |  | | + |  | |  | |  | |
| Plantaginaceae | PH | **Veronica kaiseri* Täckh. |  | | + | |  | |  | |  | |  | | + | |  |  | |  | |  | |
| Ranunculaceae | A | *Delphinium bovei* Decne. |  | | + | |  | |  | |  | |  | | + | |  |  | |  | |  | |
| Ranunculaceae | A | **Nigella arvensis* L*.* subsp. *negevensis* (Zohary) Greuter & Burdet |  | | + | |  | |  | |  | |  | | + | |  |  | |  | |  | |
| Rutaceae | S | *Haplophyllum poorei* C. C. Towns. subsp. *negevensis* Zohary & Danin |  | | + | |  | |  | |  | |  | | + | |  |  | |  | |  | |
| Solanaceae | S | *Lycium schweinfurthii* Dammer var. *aschersohnii* (Dammer)Feinbrun | + | |  | |  | |  | |  | |  | | + | |  |  | |  | |  | |
| Tamaricaceae | S | **Reaumuria hirtella* Jaub. & Spach var. *brachylepis* Zohary & Danin |  | | + | |  | |  | |  | |  | | +? | |  |  | |  | |  | |
| Zygophyllaceae | S | *Fagonia mollis* Delile var. *hispida* Zohary |  | | + | |  | |  | |  | |  | | + | | + |  | |  | |  | |
| Egypt + two countries | | | | | | | | | | | | | | | | | | | | | | | |
| Acanthaceae | PH | *Blepharis attenuata* Napper | + | |  | |  | |  | |  | |  | | + | |  |  | | + | |  | |
| Amaranthaceae | S | *Anabsis syriaca* Iljin var*. syriaca* |  | | + | |  | |  | |  | |  | | + | | + |  | |  | |  | |
| Amaranthaceae | A | *Saltia papposa (*Forssk.) Moq. |  | | + | |  | |  | |  | |  | |  | |  | + | |  | | + | |
| Amaranthaceae | S | *Haloxylon negevensis* (Iljin & Zohary) L.Boulos |  | | + | |  | |  | |  | |  | | + | |  |  | | + | |  | |
| Amaryllidaceae | PH | *Allium decaisnei* C.Presl |  | | + | |  | |  | |  | |  | | + | |  |  | | + | |  | |
| Amaryllidaceae | PH | **Allium artemisietorum* Eig & Feinbrun | + | |  | |  | |  | |  | |  | | + | |  |  | | + | |  | |
| Amaryllidaceae | PH | *Allium desertorum* Forssk. | + | |  | |  | |  | |  | |  | | + | |  |  | | + | |  | |
| Apiaceae | A | *Pimpinella cretica* Poir. var*. petraea (*Nábělek) Zohary |  | | + | |  | |  | |  | |  | | + | |  |  | | + | |  | |
| Apocynaceae | PH | **Apteranthes europaea (*Guss.) Murb. var*. judaica (*Zohary) Plowes |  | | + | |  | |  | |  | |  | | + | |  |  | | + | |  | |
| Araceae | PH | *Biarum olivieri* Blume | + | |  | |  | |  | |  | |  | | + | |  |  | | + | |  | |
| Asparagaceae | PH | *Muscari longipes* Boiss. subsp. *negevense* (Feinbrun & Danin) Hosni |  | | + | |  | |  | |  | |  | | + | |  |  | | + | |  | |
| Asparagaceae | PH | *Bellevalia desertorum* Eig & Feinbrun | + | |  | |  | |  | |  | |  | | + | |  |  | | + | |  | |
| Asparagaceae | PH | *Bellevalia eigii* Feinbrun | + | |  | |  | |  | |  | |  | | + | |  |  | | + | |  | |
| Asparagaceae | PH | *Bellevalia zoharyi* Feinbrun |  | | + | |  | |  | |  | |  | | + | |  |  | | + | |  | |
| Asparagaceae | PH | **Muscari bicolor* Boiss. | + | |  | |  | |  | |  | |  | | + | | + |  | |  | |  | |
| Asteraceae | A | **Anthemis leucanthemifolia*Boiss. & C.I.Blanche |  | | + | |  | |  | |  | |  | | + | | + |  | |  | |  | |
| Asteraceae | A | **Ifloga spicata* (Forssk.) Sch. Bip. subsp. *albescens* Chrtek |  | | + | |  | |  | |  | |  | | + | |  |  | | + | |  | |
| Asteraceae | PH | *Phagnalon nitidum* Fresen. |  | | + | |  | |  | |  | |  | | + | |  |  | | + | |  | |
| Asteraceae | A | **Centaurea lanulata* Eig |  | | + | |  | |  | |  | |  | | + | |  |  | | + | |  | |
| Asteraceae | A | **Carthamus tenuis* (Boiss. & Blanche) Bornm. subsp. *foliosus* (Boiss.) Hanelt + Cyperus | + | |  | |  | |  | |  | |  | | + | | + |  | |  | |  | |
| Asteraceae | A | **Senecio glaucus*L. subsp.*glaucus* | + | |  | |  | |  | |  | |  | | + | | + |  | |  | |  | |
| Boraginaceae | PH | *Podonosma galalensis* Schweinf. ex Boiss. | + | |  | |  | |  | |  | |  | | + | |  |  | | + | |  | |
| Brassicaceae | A | *Isatis microcarpa* J.Gay ex Boiss*.* var*. blephrocarpus* Asch. | + | |  | |  | |  | |  | |  | | + | |  |  | | + | |  | |
| Brassicaceae | A | *Nasturtiopsis coronopifolia* (Desf.) Boiss. subsp. *arabica* (Boiss.) Greuter & Burdet | + | |  | |  | |  | |  | |  | | + | |  |  | | + | |  | |
| Caryophyllaceae | A | *Eremogone sinaica* (Boiss.) Dillenb. & Kadereit. |  | | + | |  | |  | |  | |  | | + | |  |  | | + | |  | |
| Caryophyllaceae | PH | *Petrorhagia arabica* (Boiss.) P.W.Ball & Heywood |  | | + | |  | |  | |  | |  | | + | |  |  | | + | |  | |
| Caryophyllaceae | A | **Silene conoidea* L. var. *obcordata* Boiss. | + | |  | |  | |  | |  | |  | | + | | + |  | |  | |  | |
| Cistaceae | S | **Helianthemum ventosum* Boiss. | + | |  | |  | |  | |  | |  | | + | |  |  | | + | |  | |
| Euphorbiaceae | S | **Euphorbia erinacea* Boiss. & Kotschy |  | | + | |  | |  | |  | |  | | + | | + |  | |  | |  | |
| Fabaceae | PH | *Astragalus amalecitanus* Boiss. |  | | + | |  | |  | |  | |  | | + | |  |  | | + | |  | |
| Fabaceae | PH | *Astragalus camelorum* Barbey |  | | + | |  | |  | |  | |  | |  | | + |  | | + | |  | |
| Iridaceae | PH | *Iris mariae* Barbey |  | | + | |  | |  | |  | |  | | + | |  |  | | + | |  | |
| Lamiaceae | S. | **Teucrium jordanicum* (Danin) Faried var.  *jordanicum* |  | | + | |  | |  | |  | |  | | + | |  |  | | + | |  | |
| Lamiaceae | PH | *Micromeria sinaica* Benth. |  | | + | |  | |  | |  | |  | | + | |  |  | | + | |  | |
| Lamiaceae | S | *Thymus bovei* Benth. | + | |  | |  | |  | |  | |  | | + | |  |  | | + | |  | |
| Papaveraceae | A | *Hypecoum aegyptiacum* (Forssk.) Asch. & Schweinf. | + | |  | |  | |  | |  | |  | | + | |  |  | | + | |  | |
| Papaveraceae | A | **Papaver humile* Fedde subsp. *humile* | + | |  | |  | |  | |  | |  | | + | |  |  | | + | |  | |
| Plantaginaceae | PH | *Kickxia floribunda* (Boiss.) Täckh. & Boulos | + | |  | |  | |  | |  | |  | | + | |  |  | | + | |  | |
| Plantaginaceae | PH | *Linaria joppensis* Bornm. | + | |  | |  | |  | |  | |  | | + | |  |  | | + | |  | |
| Rubiaceae | PH | *Galium sinaicum* (Delile ex Decne.) Boiss. |  | | + | |  | |  | |  | |  | | + | |  |  | | + | |  | |
| Solanaceae | S | *Withania obtusifolia* Täckh. | + | |  | |  | |  | |  | |  | | + | |  |  | | + | |  | |
| Tamaricaceae | S | **Reaumuria hirtella* var. *palaestina* (Boiss.) Zohary & Danin | + | |  | |  | |  | |  | |  | | + | | + |  | |  | |  | |
| Tamaricaceae | S | *Reaumuria negevensis* Zohary & Danin |  | | + | |  | |  | |  | |  | | + | |  |  | | + | |  | |
| Egypt + three countries | | | | | | | | | | | | | | | | | | | | | | | |
| Amaryllidaceae | PH | **Allium rothii* Zucc. |  | | + | |  | |  | |  | |  | | + | | + |  | | + | |  | |
| Apiaceae | PH | **Astomaea seselifolia* (DC.) Rauschert |  | | + | |  | |  | |  | |  | | + | | + |  | | + | |  | |
| Asparagaceae | PH | **Drimia palaestina* M.B.Crespo, Mart.-Azorín & M.Á.Alonso | + | |  | |  | |  | |  | |  | | + | | + |  | | + | |  | |
| Asparagaceae | PH | **Bellevalia warburgii* Feinbrun |  | | + | |  | |  | |  | |  | | + | | + |  | | + | |  | |
| Asparagaceae | PH | **Prospero hanburyi* (Baker) Speta |  | | + | |  | |  | |  | |  | | + | | + |  | | + | |  | |
| Asteraceae | PH | **Carlina curetum*Helder subsp*. orientalis*Meusel & A.Kastner |  | | + | |  | |  | |  | |  | | + | | + |  | | + | |  | |
| Asteraceae | PH | **Centaurea postii* Boiss. | + | |  | |  | |  | |  | |  | | + | | + |  | | + | |  | |
| Asteraceae | A | **Centaurea procurrens* Sieber ex Spreng. |  | | + | |  | |  | |  | |  | | + | | + |  | | + | |  | |
| Asteraceae | A | **Crepis aculeata* (DC.) Boiss. |  | | + | |  | |  | |  | |  | | + | | + |  | | + | |  | |
| Asteraceae | PH | *Onopordum alexandrinum* Boiss. | + | |  | |  | |  | |  | |  | | + | | + |  | | + | |  | |
| Boraginaceae | PH | **Alkanna strigosa* Boiss. & Hohen. |  | | + | |  | |  | |  | |  | | + | | + |  | | + | |  | |
| Boraginaceae | PH | **Heliotropium rotundifolium* Sieber ex Lehm. | + | |  | |  | |  | |  | |  | | + | | + |  | | + | |  | |
| Brassicaceae | A | **Erucaria rostrata* (Boiss.) A.W. Hill ex Greuter & Burdet. |  | | + | |  | |  | |  | |  | | + | | + |  | | + | |  | |
| Brassicaceae | A | **Ricotia lunaria* (L.) DC. |  | | + | |  | |  | |  | |  | | + | | + |  | | + | |  | |
| Campanulaceae | PH | **Asyneuma rigidum* subsp. *sinaca* (A.DC.) Damboldt |  | | + | |  | |  | |  | |  | | + | | + |  | | + | |  | |
| Campanulaceae | A | **Campanula sulphurea* Boiss. | + | |  | |  | |  | |  | |  | | + | | + |  | | + | |  | |
| Caryophyllaceae | PH | **Bolanthus hirsutus* (Labill.) Barkoudah var. *alpinus* (Boiss.) Barkoudah | + | |  | |  | |  | |  | |  | | + | | + |  | | + | |  | |
| Caryophyllaceae | A | **Silene palaestina* Boiss. |  | | + | |  | |  | |  | |  | | + | | + |  | | + | |  | |
| Crassulaceae | PH | **Rosularia lineata* (Boiss.) A.Berger |  | | + | |  | |  | |  | |  | | + | | + |  | | + | |  | |
| Cucurbitaceae | PH | **Bryonia syriaca* Boiss. |  | | + | |  | |  | |  | |  | | + | | + |  | | + | |  | |
| Convolvullaceae | PH | **Convolvulus palaestinus*  Boiss. |  | | + | |  | |  | |  | |  | | + | | + |  | | + | |  | |
| Fabaceae | A | *Trifolium philistaeum* Zohary |  | | + | |  | |  | |  | |  | | + | | + |  | | + | |  | |
| Fabaceae | A | **Astragalus palaestinus* Eig |  | | + | |  | |  | |  | |  | | + | | + |  | | + | |  | |
| Fabaceae | PH | **Astragalus sanctus* Boiss. |  | | + | |  | |  | |  | |  | | + | | + |  | | + | |  | |
| Fabaceae | A | *Lupinus palaestinus* Boiss. |  | | + | |  | |  | |  | |  | | + | | + |  | | + | |  | |
| Fabaceae | A | **Trifolium dichroanthum* Boiss. |  | | + | |  | |  | |  | |  | | + | | + |  | | + | |  | |
| Fabaceae | A | **Trigonella arabica* Delile |  | | + | |  | |  | |  | |  | | + | | + |  | | + | |  | |
| Fabaceae | A | **Trigonella schlumbergeri* Boiss. |  | | + | |  | |  | |  | |  | | + | | + |  | | + | |  | |
| Poaceae | A | *Aegilops longissima* Schweinf. & Muschl. | + | |  | |  | |  | |  | |  | | + | | + |  | | + | |  | |
| Poaceae | A | **Trisetaria koelerioides* (Bornm. & Hack.) Melderis | + | |  | |  | |  | |  | |  | | + | | + |  | | + | |  | |
| Resedaceae | A | *Reseda stenostachya* Boiss. |  | | + | |  | |  | |  | |  | | + | | + |  | | + | |  | |
| Scrophulariaceae | PH | **Verbascum eremobium* Murb. |  | | + | |  | |  | |  | |  | | + | | + |  | | + | |  | |
| Scrophulariaceae | PH | *Verbascum fruticulosum*Post |  | | + | |  | |  | |  | |  | | + | | + |  | | + | |  | |
| Zygophyllaceae | S | *Zygophyllum dumosum* Boiss. | + | |  | |  | |  | |  | |  | | + | | + |  | | + | |  | |
| Papaveraceae | A | **Hypecoum dimidiatum* Delile |  | | + | |  | |  | |  | |  | | + | | + |  | | + | |  | |
| (IV) East Mediterranean and Arabian Peninsula extensions | | | | | | | | | | | | | | | | | | | | | | | |
| Egypt + one country | | | | | | | | | | | | | | | | | | | | | | | |
| Apiaceae | PH | *Pycnocycla tomentosa* Decne. |  | | + | |  | |  | |  | |  | |  | |  | + | |  | |  | |
| Brassicaceae | A | **Crucihimalaya kneuckeri* (Bornm.) Al-Shehbaz, O'Kane & R.A.Price | + | |  | |  | |  | |  | |  | |  | |  | + | |  | |  | |
| Gentianaceae | A | *Centaurium malzacianum* Maire |  | | + | |  | |  | |  | |  | |  | |  | + | |  | |  | |
| Lamiaceae | PH | **Mentha longifolia* (L.) L. var. *schimperi* (Briq.) Briq. |  | | + | |  | |  | |  | |  | |  | |  | + | |  | |  | |
| Caryophyllaceae | PH | *Silene schimperiana* Boiss. |  | | + | |  | |  | |  | |  | |  | |  |  | |  | | + | |
| Zygophyllaceae | PH | **Zygophyllum propinquum Decne.*subsp. *migahidii (*Hadidi) Jac.Thomas & Chaudhary | + | |  | |  | |  | |  | |  | |  | |  | + | |  | |  | |
| Egypt + two countries | | | | | | | | | | | | | | | | | | | | | | | |
| Asteraceae | A | *Anthemis induraa* Delile | + | |  | |  | |  | |  | |  | | + | |  | + | |  | |  | |
| Asteraceae | A | *Picris sulphurea* Delile | + | |  | |  | |  | |  | |  | |  | |  | + | |  | | + | |
| Lamiaceae | S | **Lavandula atriplicifolia* Benth. | + | |  | |  | |  | |  | |  | |  | |  | + | |  | | + | |
| Lamiaceae | PH | *Thymus decussatus Benth.* |  | | + | |  | |  | |  | |  | | + | |  | + | |  | |  | |
| Egypt + three countries | | | | | | | | | | | | | | | | | | | | | | | |
| Amaryllidaceae | PH | *Allium sinaiticum* Boiss. |  | | + | |  | |  | |  | |  | | + | |  | + | | + | |  | |
| Apiaceae | PH | *Ferula sinaica* Boiss. |  | | + | |  | |  | |  | |  | | + | |  | + | | + | |  | |
| Apocynaceae | PH | *Gomphocarpus sinaicus* Boiss. | + | |  | |  | |  | |  | |  | | + | |  | + | |  | | + | |
| Asteraceae | PH | *Echinops glaberrimus*DC. | + | |  | |  | |  | |  | |  | | + | |  | + | | + | |  | |
| Asteraceae | PH | *Anthemis scrobicularis* Yavin |  | | + | |  | |  | |  | |  | | + | |  | + | | + | |  | |
| Asteraceae | PH | **Atractylis mernephthae* Asch. & Schweinf. & Letourn. | + | |  | |  | |  | |  | |  | | + | |  | + | | + | |  | |
| Asteraceae | S | *Centaurea scoparia* Sieber ex Spreng. | + | |  | |  | |  | |  | |  | | + | |  | + | | + | |  | |
| Asteraceae | PH | *Iphiona mucronata* (Forssk.) Asch. & Schweinf. | + | |  | |  | |  | |  | |  | | + | |  | + | | + | |  | |
| Asteraceae | PH | *Phagnalon barbeyanum* Asch. & Schweinf. | + | |  | |  | |  | |  | |  | | + | |  | + | | + | |  | |
| Asteraceae | PH | *Phagnalon sinaicum* Bornm. & Kneuck. |  | | + | |  | |  | |  | |  | | + | |  | + | |  | | + | |
| Asteraceae | PH | *Tanacetum sinaicum* (Fresen.) Delile ex K.Bremer & Humphries |  | | + | |  | |  | |  | |  | | + | |  | + | | + | |  | |
| Brassicaceae | PH | *Matthiola arabica* Boiss. |  | | + | |  | |  | |  | |  | | + | |  | + | | + | |  | |
| Campanulaceae | PH | *Campanula dulcis* Decne. |  | | + | |  | |  | |  | |  | | + | |  | + | | + | |  | |
| Caprifoliaceae | S | *Pterocephalus sanctus* Decne. |  | | + | |  | |  | |  | |  | | + | |  | + | | + | |  | |
| Caryophyllaceae | PH | *Dianthus sinaicus* Boiss. |  | | + | |  | |  | |  | |  | | + | |  | + | | + | |  | |
| Caryophyllaceae | PH | **Paronychia sinaica* Fresen. | + | |  | |  | |  | |  | |  | | + | |  | + | | + | |  | |
| Caryophyllaceae | A | **Silene hussonii* Boiss. |  | | + | |  | |  | |  | |  | | + | |  | + | | + | |  | |
| Colchicaceae | PH | *Colchicum guessfeldtianum* Asch. & Schweinf. | + | |  | |  | |  | |  | |  | | + | |  | + | | + | |  | |
| Convolvulaceae | PH | *Convolvulus spicatus*Peter ex Hallier f. |  | | + | |  | |  | |  | |  | | + | |  | + | | + | |  | |
| Fabaceae | PH | *Astragalus fruticosus* Forssk. | + | |  | |  | |  | |  | |  | | + | |  | + | | + | |  | |
| Fabaceae | A | *Astragalus intercedens* Sam. ex Rech.f. |  | | + | |  | |  | |  | |  | | + | |  | + | | + | |  | |
| Fabaceae | PH | *Bituminaria flaccida* (Nábelek) Greuter |  | | + | |  | |  | |  | |  | | + | |  | + | | + | |  | |
| Hypericaceae | PH | *Hypericum sinaicum* Hochst & Steud. ex Boiss. |  | | + | |  | |  | |  | |  | | + | |  | + | | + | |  | |
| Lamiaceae | S | *Salvia deserti* Decne. |  | | + | |  | |  | |  | |  | | + | |  | + | | + | |  | |
| Lamiaceae | PH | *Stachys aegyptiaca* Pers. | + | |  | |  | |  | |  | |  | | + | |  | + | | + | |  | |
| Lamiaceae | S | *Teucrium leucocladum* Boiss. var. *leucocladum* | + | |  | |  | |  | |  | |  | | + | |  | + | | + | |  | |
| Polygalaceae | S | *Polygala sinaica* Botsch. var. *glabrescens* (Zohary) Boulos | + | |  | |  | |  | |  | |  | | + | |  | + | | + | |  | |
| Scrophulariaceae | PH | *Verbascum schimperianum* Boiss. |  | | + | |  | |  | |  | |  | | + | |  | + | | + | |  | |
| (V) Sudanian and Arabian Peninsula extensions | | | | | | | | | | | | | | | | | | | | | | | |
| Anacardiaceae | S | **Searsia flexicaulis* (Baker) Moffett | + | |  | | + | |  | |  | |  | |  | |  |  | |  | | + | |
| Amaryllidaceae | S | **Pancratium tortuosum* Herb. | + | |  | | + | |  | |  | | + | |  | |  | + | |  | |  | |
| Asteraceae | PH | *Echinops hussonii* Boiss. | + | |  | | + | |  | |  | |  | |  | |  | + | |  | | + | |
| Fabaceae | S | *Taverniera aegyptiaca* Boiss. | + | |  | | + | |  | |  | | + | |  | |  | + | |  | |  | |
| Zygophyllaceae | S | **Tribulus spurius* Kralik | + | |  | | + | |  | |  | |  | |  | |  | + | |  | | + | |
| (VI) North African and Arabian Peninsula extensions | | | | | | | | | | | | | | | | | | | | | | | |
| Asteraceae | PH | *Echinops galalensis* Schweinf. | + | |  | |  | | + | |  | |  | |  | |  | + | |  | |  | |
| Boraginaceae | A | **Echium longifolium* Delile | + | |  | |  | | + | |  | |  | | + | |  |  | | + | |  | |
| Brassicaceae | A | **Erucaria microcarpa* Boiss. | + | |  | |  | | + | |  | |  | | + | |  |  | | + | |  | |
| (VII) North African and Sudanian extensions | | | | | | | | | | | | | | | | | | | | | | | |
| Poaceae | A | *Stipagrostis shawii* (H.Scholz) H.Scholz | + | |  | | + | | + | |  | |  | |  | |  |  | |  | |  | |
| (VIII) Widely distributed, multiple extensions | | | | | | | | | | | | | | | | | | | | | | | |
| Apocynaceae | PH | **Caudanthera sinaica* (Decne.) Plowes |  | | + | | + | |  | |  | |  | | + | |  | + | |  | |  | |
