# Supplementary material for: New perspectives, additions, and amendments to plant endemism in a North African flora
Source: Bot Stud. 2024 Jul 16;65:21. doi: 10.1186/s40529-024-00428-w (PMC11252113; doi:10.1186/s40529-024-00428-w)
Supplement: Supplementary file 5 — Supplementary Material 5. [file 40529_2024_428_MOESM5_ESM.doc]

**Supplementary Table 3A** Chao-Jaccard similarity index (C-J) and the shared endemic taxa (between parentheses) among the 14 OGUs (for description of OGUs, see Figure 1)

| OGUs | S | Di | Ms | Mm | Nv | Nn | Di | Ol | On | Dg | Ge | Ra | Da |
| --- | --- | --- | --- | --- | --- | --- | --- | --- | --- | --- | --- | --- | --- |
| S |  |  |  |  |  |  |  |  |  |  |  |  |  |
| Di | 0.7 (5) |  |  |  |  |  |  |  |  |  |  |  |  |
| Ms | 0.3 (1) | 0.5 (1) |  |  |  |  |  |  |  |  |  |  |  |
| Mm | 0.3 (1) | 0.5 (1) | 0.7 (3) |  |  |  |  |  |  |  |  |  |  |
| Nv | 0.4 (2) | 0.6 (2) | 0.5 (1) | 0.9 (5) |  |  |  |  |  |  |  |  |  |
| Nn | 0.3 (1) | 0.5 (1) | 0.5 (1) | 0.5 (1) | 0.3 (1) |  |  |  |  |  |  |  |  |
| Dl | 0.4 (1) | 0.6 (1) | 0.5 (1) | 0.9 (2) | 0.8 (2) | 0.5 (1) |  |  |  |  |  |  |  |
| Ol | 0.4 (1) | 0.6 (1) | 0.5 (1) | 0.5 (1) | 0.3 (1) | 0.5 (1) | 0.6 (1) |  |  |  |  |  |  |
| On | 0.3 (1) | 0.5 (1) | 0.5 (1) | 0.5 (1) | 0.3 (1) | 0.5 (1) | 0.5 (1) | 0.5 (1) |  |  |  |  |  |
| Dg | 0.3 (1) | 0.6 (2) | 0.5 (1) | 0.6 (2) | 0.4 (2) | 0.5 (1) | 0.5 (1) | 0.6 (1) | 0.5 (1) |  |  |  |  |
| Ge | 0.3 (1) | 0.5 (1) | 0.6 (1) | 0.5 (1) | 0.3 (1) | 0.5 (1) | 0.5 (1) | 0.5 (1) | 0.5 (1) | 0.5 (1) |  |  |  |
| Ra | 0.3 (1) | 0.5 (1) | 0.5 (1) | 0.5 (1) | 0.3 (1) | 0.5 (1) | 0.5 (1) | 0.5 (1) | 0.5 (1) | 0.5 (1) | 0.9 (5) |  |  |
| Da | 0.3 (3) | 0.6 (2) | 0.5 (1) | 0.6 (2) | 0.6 (3) | 0.5 (1) | 0.5 (1) | 0.5 (1) | 0.5 (1) | 0.7 (2) | 0.3 (1) | 0.3 (1) |  |
| Rz | 0.5 (7) | 0.8 (3) | 0.5 (1) | 0.5 (1) | 0.4 (2) | 0.5 (1) | 0.5 (1) | 0.5 (1) | 0.5 (1) | 0.5 (1) | 0.3 (1) | 0.3 (1) | 0.7 (2) |

**Supplementary Table 3B** Jaccard’s similarity index between near-endemic taxa recorded in previous studies

| Authors | Jaccard’s Measure | | | |
| --- | --- | --- | --- | --- |
|  | Boulos (2009) | Hosni *et al.* (2013) | Shaltout *et al.* (2018) | POWO* |
| Boulos (2009) |  |  |  |  |
| Hosni *et al.* (2013) | 0.54 |  |  |  |
| Shaltout *et al.* (2018) | 0.66 | 0.44 |  |  |
| POWO* | 0.44 | 0.42 | 0.35 |  |
| Present | 0.42 | 0.44 | 0.34 | 0.89 |

*= ([http://www.plantsoftheworldonline.org](http://www.plantsoftheworldonline.org/)
