# Supplementary material for: New perspectives, additions, and amendments to plant endemism in a North African flora
Source: Bot Stud. 2024 Jul 16;65:21. doi: 10.1186/s40529-024-00428-w (PMC11252113; doi:10.1186/s40529-024-00428-w)
Supplement: Supplementary file 6 — Supplementary Material 6. [file 40529_2024_428_MOESM6_ESM.doc]

**Supplementary Table 4** Distribution of near-endemic taxa in the different OGUs of Egypt; see Figure (1) for full names of OGUs abbreviations.

| **Taxa** | **Taxa** | **OGUs** |
| --- | --- | --- |
| **Taxa recorded in more than 4 OGUs** |  |  |
| Amaryllidaceae | *Allium tel-avivense* Eig | Mm, Ms, Dg, Di, S |
| Resedaceae | *Reseda pruinosa* Delile | Mm, Di, Dg, Ol, S, Ge |
| **Taxa recorded in four OGUs** |  |  |
| Apiaceae | *Pycnocycla tomentosa* Decne. | Di, S, Rq, Rz |
| Asteraceae | *Ifloga spicata (Forssk.)* Sch. Bip. subsp.*albescens* Chrtek | Dg, Da, S, Ge |
| Asteraceae | *Picris sulphurea*Delile | Nv, Da, S, Ol |
| Asteraceae | *Phagnalon barbeyanum*Asch. & Schweinf*.* | Dg, Ra, Di, S |
| Brassicaceae | *Isatis microcarpa* J.Gay ex Boiss. var. *blephrocarpus* Asch. | Ms, Di, Dg, S |
| Brassicaceae | *Isatis microcarpa*J.Gay ex Boiss. var. *microcarpus* | Ms, Di, Dg, S |
| Caryophyllaceae | *Petrorhagia arabica*(Boiss.) P.W.Ball & Heywood | Di, S, Rq, Rz |
| Fabaceae | *Astragalus hispidulus* DC. | Ol, Mm, Ms, S |
| Lamiaceae | *Stachys aegyptiaca*Pers. | Dg, Di, S, Ra |
| Plantaginaceae | *Veronica catenata*subsp.*pseudocatenata*Chrtek & Osb.-Kos. | Mm, Dg, Nv, Ol |
| Plantaginaceae | *Kickxia floribunda (*Boiss.) Täckh. & Boulos | Mm, Ms, Di, S |
| Zygophyllaceae | *Fagonia mollis* Delile var. *hispida* Zohary | Di, S, Rq, Rz |
| **Taxa recorded in three OGUs** |  |  |
| Amaryllidaceae | *Allium artemisietorum* Eig & Feinbrun | Mm, Dg, Di |
| Amaryllidaceae | *Allium desertorum*Forssk. | Mm, Dg, Di |
| Amaryllidaceae | *Pancratium tortuosum*Herb. | Da, Ra, Ge |
| Apiaceae | *Foeniculum piperitum (*Ucria) C.Presl | Mm, Ms, Nv |
| Apocynaceae | *Gomphocarpus sinaicus* Boiss. | Di, S, Dg |
| Araceae | *Biarum olivieri*Blume | Mm, Ms, Di |
| Asparagaceae | *Drimia palaestina*M.B.Crespo, Mart.-Azorín & M.Á.Alonso | Ms, Dg, Di |
| Asteraceae | *Anthemis indurata*Delile | Mm, Ms, Di |
| Asteraceae | *Centaurea glomerata*Vahl | Mm, Dl, Di |
| Asteraceae | *Echinops hussonii*Boiss. | Da, Ge, Ra |
| Asteraceae | *Senecio glaucus*L.subsp*. glaucus* | Dg, Ms, S |
| Asteraceae | *Centaurea scoparia*Sieber ex Spreng. | Dg, Ra, S |
| Asteraceae | *Echinops glaberrimus*DC. | Dg, S, Rq |
| Asteraceae | *Iphiona mucronata (*Forssk.) Asch. & Schweinf. | Dg, Di, S |
| Asteraceae | *Onopordum alexandrinum*Boiss. | Mm, Ms, S |
| Boraginaceae | *Heliotropium rotundifolium*Sieber ex Lehm. | Dg, Di, S |
| Brassicaceae | *Pseuderucaria clavata (*Boiss. & Reut.) O. E. Schulz subsp*. clavata* | Dg, Di, S |
| Caryophyllaceae | *Paronychia sinaica* Fresen. | Dg, Di, S |
| Colchicaceae | *Colchicum guessfeldtianum*Asch. & Schweinf. | Dg, Da, S |
| Fabaceae | *Astragalus camelorum*Barbey | Ms, Di, Rz |
| Fabaceae | *Lotus hebranicus* Hochst. ex Brand | Dg, S, Ra |
| Lamiaceae | *Lavandula atriplicifolia*Benth | Da, Ge, Ra |
| Lamiaceae | *Micromeria sinaica*Benth. | Di, S, Rq |
| Lamiaceae | *Teucrium decaisnei*C. Presl | Dg, Di, S |
| Lamiaceae | *Salvia deserti*Decne. | Di, Dg, S |
| Lamiaceae | *Teucrium leucocladum* Boiss. var. *leucocladum* | Di, S, Ra |
| Papaveraceae | *Hypecoum aegyptiacum (*Forssk.) Asch. & Schweinf. | Mm, Ms, Di |
| Plantaginaceae | *Veronica scardica*subsp.*africana*Chrtek & Osb.-Kos. | Nn, Ol, On |
| Scrophulariaceae | *Verbascum letourneuxii*Asch. | Mm, Ms, Di |
| Tamaricaceae | *Reaumuria negevensis* Zohary & Danin | Di, S, Rq |
| **Taxa recorded in two OGUs** |  |  |
| Acanthaceae | *Blepharis attenuata* Napper | Dg, S |
| Amaryllidaceae | *Allium curtum* subsp. *palaestinum* Feinbrun | Ms, Di |
| Amaryllidaceae | *Allium decaisnei* C.Presl | Di, S |
| Amaryllidaceae | *Allium papillare*Boiss. | Di, S |
| Amaryllidaceae | *Allium sinaiticum* Boiss. | Di, S |
| Amaryllidaceae | *Allium rothii*Zucc. | Di, S |
| Anacardiaceae | *Searsia flexicaulis* (Baker) Moffett | Ge, Ra |
| Apiaceae | *Ferula sinaica* Boiss. | Di, S |
| Apiaceae | *Ferula marmarica* Asch. & Taub. | Mm, Dl |
| Apiaceae | *Astomaea seselifolia*(DC.) Rauschert | Di, S |
| Apocynaceae | *Caudanthera sinaica* (Decne.) Plowes | Di, S |
| Arecaceae | *Medemia argun* (Mart.) Württemb. ex H.Wendl. | Dn, On |
| Asparagaceae | *Muscari eburneum* (Eig & Feinbrun) D.C.Stuart | Ms, Di |
| Asparagaceae | *Muscari longistylum*(Täckh. & Boulos) Hosni | Ms, Di |
| Asparagaceae | *Bellevalia desertorum* Eig & Feinbrun | Ms, Di |
| Asparagaceae | *Bellevalia eigii* Feinbrun | Ms, Di |
| Asparagaceae | *Muscari bicolor* Boiss. | Ms, Di |
| Asparagaceae | *Bellevalia warburgii* Feinbrun | Di, S |
| Asteraceae | *Atractylis carduus* (Forssk.) C.Chr. var. *glabrescens* (Boiss.) Täckh. & Boulos | Ms, S |
| Asteraceae | *Carthamus mareoticus* Delile | Mm, Dl |
| Asteraceae | *Centaurea lanulata* Eig | Ms, Di |
| Asteraceae | *Echinops glaberrimus* DC. | Dg, S |
| Asteraceae | *Phagnalon nitidum* Fresen. | Dg, S |
| Asteraceae | *Atractylis mernephthae* Asch. & Schweinf. & Letourn. | Dg, S |
| Asteraceae | *Centaurea procurrens* Sieber ex Spreng. | Ms, S |
| Asteraceae | *Carlina curetum* Helder subsp*. orientalis*Meusel & A.Kastner | Mm, Dg |
| Boraginaceae | *Alkanna strigosa* Boiss. & Hohen. | Ms, S |
| Brassicaceae | *Enarthrocarpus pterocarpus* (Pers.) DC. | Mm, Nv |
| Brassicaceae | *Nasturtiopsis coronopifolia* (Desf.) Boiss. subsp. *arabica* (Boiss.) Greuter & Burdet | Di, S |
| Brassicaceae | *Erucaria rostrata* (Boiss.) A.W. Hill ex Greuter & Burdet. | Di, S |
| Brassicaceae | *Matthiola arabica* Boiss. | Di, S |
| Brassicaceae | *Zilla spinosa*(L.) Prantl subsp. *biparmata* (O.E.Schulz) Maire & Weiller | Mm, Dg |
| Campanulaceae | *Campanula sulphurea* Boiss. | Dg, S |
| Caryophyllaceae | *Eremogone sinaica* (Boiss.) Dillenb. & Kadereit | Di, S |
| Caryophyllaceae | *Silene biappendiculata* Ehrh. ex Rohrb. | Mm, Ms |
| Caryophyllaceae | *Silene schimperiana* Boiss. | Di, S |
| Caryophyllaceae | *Bolanthus hirsutus* (Labill.) Barkoudah var. *alpinus* (Boiss.) Barkoudah | Di, S |
| Caryophyllaceae | *Dianthus sinaicus* Boiss. | Di, S |
| Cistaceae | *Helianthemum ventosum* Boiss. | Di, S |
| Convolvullaceae | *Convolvulus palaestinus* Boiss. | Di, S |
| Convolvullaceae | *Convolvulus spicatus* Peter ex Hallier f. | Di, S |
| Crassulaceae | *Rosularia lineata* (Boiss.) A.Berger | Di, S |
| Cyperaceae | *Cyperus microbolbos* C.B.Clarke | Ge, Ra |
| Euphorbiaceae | *Euphorbia erinacea* Boiss. & Kotschy | Di, S |
| Euphorbiaceae | *Euphorbia parvula* Delile | Mm, Ms |
| Fabaceae | *Lotus nubicus* Hochst. ex Baker | Ra, Ge |
| Fabaceae | *Lotus polyphyllos*E.D.Clarke | Mm, Dl |
| Fabaceae | *Trifolium philistaeum* Zohary | Ms, Di |
| Fabaceae | *Astragalus fruticosus* Forssk. | Ms, Di |
| Fabaceae | *Astragalus intercedens* Sam. ex Rech.f. | Ms, Di |
| Fabaceae | *Trigonella arabica*Delile | Ms, Di |
| Iridaceae | *Iris mariae* Barbey | Ms, Di |
| Lamiaceae | *Mentha longifolia* (L.) L. var. *schimperi* (Briq.) Briq. | Di, S |
| Lamiaceae | *Origanum isthmicum* Danin | Di, S |
| Lamiaceae | *Pseudodictamnus damascenus* (Boiss.) Salmaki & Siadati | Dg, S |
| Lamiaceae | *Teucrium jordanicum* (Danin) Faried var. *jordanicum* | Di, S |
| Papaveraceae | *Hypecoum aequilobum*Viv. | Mm, Di |
| Papaveraceae | *Papaver humile* Fedde subsp. *humile* | Di, S |
| Papaveraceae | *Hypecoum dimidiatum* Delile | Ms, S |
| Plantaginaceae | *Linaria joppensis* Bornm. | Mm, Ms |
| Plumbaginaceae | *Limonium tubiflorum* (Delile) Kuntze | Mm, Dl |
| Plumbaginaceae | *Limonium zanonii*(Pamp.) Domina | Mm, Dl |
| Poaceae | *Aegilops longissima* Schweinf. & Muschl. | Mm, Ms |
| Ranunculaceae | *Delphinium bovei* Decne. | Ms, Di |
| Rubiaceae | *Galium sinaicum* (Delile ex Decne.) Boiss. | Dg, S |
| Scrophulariaceae | *Withania obtusifolia* Täckh. | S, Ge |
| Solanaceae | *Lycium schweinfurthii* Dammer var. *aschersohnii* (Dammer) Feinbrun | Mm, Ms |
| Solanaceae | *Withania obtusifolia* Täckh. | Ge, S |
| Tamaricaceae | *Reaumuria hirtella* Jaub. & Spach. var. *brachylepis* Zohary & Danin | Mm, Ms |
| Tamaricaceae | *Reaumuria hirtella* var. *palaestina* (Boiss.) Zohary & Danin | Di, S |
| Zygophyllaceae | *Tribulus spurius* Kralik | Dn, Ge |
| Zygophyllaceae | *Zygophyllum dumosum* Boiss. | Dg, S |
| **Taxa recorded in one OGUs** |  |  |
| Amaranthaceae | *Anabsis syriaca* Iljin var. *syriaca* | Di |
| Amaranthaceae | *Saltia papposa*(Forssk.) Moq. | S |
| Amaranthaceae | *Haloxylon negevensis (*Iljin & Zohary) L.Boulos | Di |
| Amaryllidaceae | *Allium barthianum* Asch. & Schweinf. | Mm |
| Amaryllidaceae | *Allium blomfieldianum*Asch. & Schweinf. | Mm |
| Apiaceae | *Bupleurum nanum*Poir. | Mm |
| Apiaceae | *Daucus syrticus*Murb. | Mm |
| Apocynaceae | *Apteranthes europaea* (Guss.) Murb. var. *judaica* (Zohary) Plowes | Di |
| Asparagaceae | *Bellevalia sessiliflora*(Viv.) Kunth | Mm |
| Asparagaceae | *Muscari longipes* Boiss*.* subsp*. negevense*(Feinbrun & Danin) Hosni | Di |
| Asparagaceae | *Bellevalia zoharyi* Feinbrun | Di |
| Asparagaceae | *Prospero hanburyi*(Baker) Speta | S |
| Asteraceae | *Atractylis boulosii*Täckh. | S |
| Asteraceae | *Carthamus glaucus*subsp.*alexandrinus*(Boiss. & Heldr.) Hanelt | Mm |
| Asteraceae | *Crepis libyca (Pamp.)* Babc*.* | Mm |
| Asteraceae | *Anthemis eliezrae*Eig | S |
| Asteraceae | *Anthemis leucanthemifolia*Boiss. & C.I.Blanche | Ms |
| Asteraceae | *Anthemis scrobicularis*Yavin | S |
| Asteraceae | *Centaurea postii Boiss.* | S |
| Asteraceae | *Crepis aculeata*(DC.) Boiss. | Ms |
| Asteraceae | *Phagnalon sinaicum*Bornm. & Kneuck. | S |
| Asteraceae | *Tanacetum sinaicum*(Fresen.) Delile ex K.Bremer & Humphries | S |
| Boraginaceae | *Nonea vivianii*DC. | Mm |
| Boraginaceae | *Podonosma galalensis*Schweinf. ex Boiss. | Dg |
| Brassicaceae | *Crucihimalaya kneuckeri*(Bornm.) Al-Shehbaz, O'Kane & R.A.Price | S |
| Brassicaceae | *Ricotia lunaria*(L.) DC. | S |
| Campanulaceae | *Asyneuma rigidum*subsp.*sinaca*(A.DC.) Damboldt | S |
| Campanulaceae | *Campanula dulcis* Decne | S |
| Caprifoliaceae | *Valerianella pterovichii* Asch. | Mm |
| Caprifoliaceae | *Pterocephalus sanctus*Decne. | S |
| Caryophyllaceae | *Herniaria cyrenaica* F.Herm. | Mm |
| Caryophyllaceae | *Silene fruticosa L.* subsp. *cyrenaica* Bég. et A. Vacc. | Mm |
| Caryophyllaceae | *Silene conoidea L.* var. *obcordata* Boiss. | Di |
| Caryophyllaceae | *Silene hussonii* Boiss. | S |
| Caryophyllaceae | *Silene palaestina*Boiss. | S |
| Cistaceae | *Helianthemum crassifolium*subsp.*sphaerocalyx*(Gauba & Janch.) Maire | Mm |
| Cucurbitaceae | *Coccinia abyssinica*(Lam.) Cogn. | Ge |
| Cucurbitaceae | *Bryonia syriaca*Boiss. | S |
| Fabaceae | *Ebenus armitagei*Schweinf. & Taub. | Mm |
| Fabaceae | *Astragalus amalecitanus* Boiss. | Di |
| Fabaceae | *Taverniera aegyptiaca*Boiss. | Ge |
| Fabaceae | *Astragalus palaestinus*Eig | S |
| Fabaceae | *Astragalus sanctus*Boiss. | Di |
| Fabaceae | *Bituminaria flaccida*(Nábelek) Greuter | S |
| Fabaceae | *Lupinus palaestinus*Boiss. | Di |
| Fabaceae | *Trifolium dichroanthum*Boiss. | S |
| Fabaceae | *Trigonella schlumbergeri*Boiss. | S |
| Gentianaceae | *Centaurium malzacianum*Maire | S |
| Hypericaceae | *Hypericum sinaicum* Hochst & Steud. ex Boiss. | S |
| Lamiaceae | *Thymus bovei*Benth. | Di |
| Lamiaceae | *Thymus decussatus*Benth. | S |
| Plantaginaceae | *Veronica anagallis-aquatica* var. *nilotica* R.Uechr. | Nn |
| Plantaginaceae | *Veronica kaiseri* Täckh. | S |
| Poaceae | *Stipagrostis shawii*(H.Scholz) H.Scholz | UW |
| Poaceae | *Trisetaria koelerioides*(Bornm. & Hack.) Melderis | S |
| Polygalaceae | *Polygala sinaica*Botsch*.* | S |
| Polygalaceae | *Polygala sinaica*Botsch. var.*glabrescens*(Zohary) Boulos | S |
| Ranunculaceae | *Nigella arvensis* L. subsp.*negevensis*(Zohary) Greuter & Burdet | Ms |
| Ranunculaceae | *Nigella arvensis*L. subsp. *taubertii*(Brand) Maire | Mm |
| Resedaceae | *Reseda lutea* L. subsp. *petrovichiana* (Müll.Arg.) Jafri | Mm |
| Resedaceae | *Reseda stenostachya* Boiss. | S |
| Rubiaceae | *Valantia columella*(Ehrenb. ex Boiss.) Bald. | Mm |
| Rutaceae | *Haplophyllum poorei* C. C. Towns. subsp. *negevensis* Zohary & Danin | S |
| Scrophulariaceae | *Verbascum fruticulosum* Post | S |
| Scrophulariaceae | *Verbascum eremobium*Murb. | S |
| Scrophulariaceae | *Verbascum schimperianum Boiss.* | S |
| Zygophyllaceae | *Zygophyllum propinquum*Decne. subsp. *migahidii* (Hadidi) Jac.Thomas & Chaudhary | Di |
