# Supplementary material for: New perspectives, additions, and amendments to plant endemism in a North African flora
Source: Bot Stud. 2024 Jul 16;65:21. doi: 10.1186/s40529-024-00428-w (PMC11252113; doi:10.1186/s40529-024-00428-w)
Supplement: Supplementary file 8 — Supplementary Material 8. [file 40529_2024_428_MOESM8_ESM.doc]

**Supplementary Table 6 Summary of variations in the estimated numbers of endemic taxa in the flora of Egypt**

| Authors | Total number of endemics enumerated | Numbers of  endemic  taxa considered | Taxa | Notes |
| --- | --- | --- | --- | --- |
| Täckholm (1974) | 55 | 11 | see Supplementary Table 4 | 8/11 now are near-endemics |
| El Hadidi and Fayed (1994/95) | 49 | 2 | *Ebenus armitagae, Centaurium malzacianum* | Near-endemics (in Libya for the former and in Arabia for the latter) |
| El Hadidi and Hosni (2000) | 6 | 3 | *Juncus bufonius* var. *subauriculata*)  *Solanum sinaicum*  *Salvia palaestina* | Near-endemics as the first two taxa are currently widely distributed |
| Boulos (2009) | 60 | 3 | *Phagnalon nitidum* (+ Palestine)  *Plantago sinaica* (+ Palestine)  *Veronica catenata* subsp. *pseudocatenata* (+ Libya) |  |
| Hosni *et al.* (2013) | 76 | 1 | *Muscari longistylum* | Its occurrence extended to Palestine, now considered as near endemic in this study |
| Abdelaal *et al.* (2018) | 48 | 0 | (No additions) |  |
| El-Khalafy *et al.* (2021) | 41 | 0 | (No additions) |  |
| Present study | 70 | 19 | (newly added); | Supplementary Table 1 |
